# Prenatal maternal stress and wheeze in children: novel insights into epigenetic regulation

**DOI:** 10.1038/srep28616

**Published:** 2016-06-28

**Authors:** Saskia Trump, Matthias Bieg, Zuguang Gu, Loreen Thürmann, Tobias Bauer, Mario Bauer, Naveed Ishaque, Stefan Röder, Lei Gu, Gunda Herberth, Christian Lawerenz, Michael Borte, Matthias Schlesner, Christoph Plass, Nicolle Diessl, Markus Eszlinger, Oliver Mücke, Horst-Dietrich Elvers, Dirk K. Wissenbach, Martin von Bergen, Carl Herrmann, Dieter Weichenhan, Rosalind J. Wright, Irina Lehmann, Roland Eils

**Affiliations:** 1 Department of Environmental Immunology, Helmholtz Centre for Environmental Research Leipzig-UFZ, Permoserstraße 15, Leipzig, 04318, Germany; 2 Division of Theoretical Bioinformatics, German Cancer Research Center (DKFZ), Im Neuenheimer Feld 280, Heidelberg, 69120, Germany; 3 Heidelberg Center for Personalized Oncology, DKFZ-HIPO, DKFZ, Im Neuenheimer Feld 267, Heidelberg, 69120, Germany; 4 Division of Newborn Medicine, Boston Children’s Hospital, Harvard Medical School, Boston, Massachusetts, USA; 5 Municipal Hospital “St. Georg” Children’s Hospital, Delitzscher Straße 141, 04129 Leipzig, Germany; 6 Division of Epigenomics and Cancer Risk Factors, German Cancer Research Center (DKFZ), Im Neuenheimer Feld 280, Heidelberg, 69120, Germany; 7 Genomics and Proteomics Core Facility, German Cancer Research Center (DKFZ), Im Neuenheimer Feld 280, Heidelberg, 69120, Germany; 8 Division of Endocrinology and Nephrology, University of Leipzig, Liebigstraße 21, Leipzig, 04103, Germany; 9 Department of Urban and Environmental Sociology, Helmholtz Centre for Environmental Research Leipzig-UFZ, Permoserstraße 15, Leipzig, 04318, Germany; 10 Department of Molecular Systems Biology, Helmholtz Centre for Environmental Research-UFZ, 04318, Leipzig, Germany; 11 Faculty of Biosciences, Pharmacy, and Psychology, University of Leipzig, 04318, Leipzig, Germany; 12 Department of Chemistry and Biosciences, Aalborg University, DK-9220 Aalborg, Denmark; 13 Institute of Pharmacy and Molecular Biotechnology, and Bioquant Center, University of Heidelberg, Im Neuenheimer Feld 267, Heidelberg, 69120, Germany; 14 Department of Pediatrics, Kravis Children’s Hospital, Mindich Child Health and Development Institute, Icahn School of Medicine at Mount Sinai, New York, NY 10029, USA; 15 Translational Lung Research Center Heidelberg (TLRC), German Center for Lung Research (DZL), University of Heidelberg, 69120, Germany

## Abstract

Psychological stress during pregnancy increases the risk of childhood wheeze and asthma. However, the transmitting mechanisms remain largely unknown. Since epigenetic alterations have emerged as a link between perturbations in the prenatal environment and an increased disease risk we used whole genome bisulfite sequencing (WGBS) to analyze changes in DNA methylation in mothers and their children related to prenatal psychosocial stress and assessed its role in the development of wheeze in the child. We evaluated genomic regions altered in their methylation level due to maternal stress based of WGBS data of 10 mother-child-pairs. These data were complemented by longitudinal targeted methylation and transcriptional analyses in children from our prospective mother-child cohort LINA for whom maternal stress and wheezing information was available (n = 443). High maternal stress was associated with an increased risk for persistent wheezing in the child until the age of 5. Both mothers and children showed genome-wide alterations in DNA-methylation specifically in enhancer elements. Deregulated neuroendocrine and neurotransmitter receptor interactions were observed in stressed mothers and their children. In children but not in mothers, calcium- and Wnt-signaling required for lung maturation in the prenatal period were epigenetically deregulated and could be linked with wheezing later in children’s life.

The psychosocial health of a pregnant woman can significantly impact the growing fetus and predispose offspring toward increased risk of developing obstructive airway diseases later in life. Although maternal stress during pregnancy has been linked to the development of repeated wheeze, asthma, or airway hyper-responsiveness^1^,^2^,^3^,^4^,^5^, the mechanisms involved remain unknown. Evidence largely from animal studies suggests that stress modulates neuroendocrine and autonomic nervous system (ANS) responses to impact immune system functioning and lung development^6^,^7^,^8^,^9^. There is growing evidence in humans that prenatal stress alters immune function in the developing fetus as indicated by perturbed stimulated cytokine responses and elevated IgE levels at birth^2^,^10^. It has been suggested that an aberrant or excessive pro-inflammatory immune response contributes to lung structure-function changes promoting the development of wheezing syndromes later in life^11^,^12^.

In the current study we assessed the perceived stress level of women during the course of their pregnancy and were able to confirm previous findings showing a positive association of the prenatal maternal stress levels with an increase in risk for the child to develop persistent wheezing. Our central aim was to assess possible biological mechanisms by which prenatal stress translates into lung dysfunction in the child.

It is hypothesized that transient effects of environmental exposure early in life, in this case prenatal maternal stress, can be preserved by epigenetic mechanisms (e.g. DNA methylation) that silence or activate disease relevant signaling pathways in a persistent way and thereby may modify disease susceptibility^13^. Such stress induced epigenetic programming leading to adverse respiratory outcomes may be influenced by a variety of pathways involved in lung development beyond the suggested aberrant immune response. There is a lack of comprehensive studies on genome-wide stress-dependent epigenetic perturbations. To fill this gap we chose a well-defined set of mothers and their newborn children to study stress-dependent DNA methylation changes in an unbiased manner by whole genome bisulfite sequencing covering every single base pair of the genome. Based on this genome-wide analysis a hypothesis was developed for the link between prenatal stress and respiratory outcomes and validated by targeted methylation and RNA expression analysis in the entire LINA cohort.

## Methods

Further information and details are available in the Supplementary Information.

### Study Design

This study is based on a subset of the prospective mother-child cohort LINA (**L**ifestyle and environmental factors and their **I**nfluence on **N**ewborns **A**llergy risk), which originally included 629 mother–child pairs (622 mothers, 629 children; 7 twin-pairs) recruited between May 2006 and December 2008 in Leipzig, Germany^14^. These analyses included 443 mother-child pairs for which information on maternal stress level, and prevalence of wheeze of the child up to the age of 5 years was available (Table 1). Wheezing phenotypes were classified according to Martinez *et al*.^15^. Information on possible confounders was obtained from questionnaires completed by mothers during pregnancy and around their child’s first birthday^16^. Study participation was voluntary, written informed consent from all participants and institutional review board approval was obtained (University of Leipzig, 046-2006, 160-2008, 160-2008, 160b/2008, 144-10-31052010, 113-11-18042011). The methods applied in this study were in accordance with the approved guidelines.

### Perceived Stress Assessment

Maternal stress was assessed by a 20-item perceived stress questionnaire (PSQ)^17^,^18^ evaluating how often certain experiences of stress (worries, tension, loss of joy, demands) occurred on a 4-point scale (1 through 4, Table E1)^17^. A total score was calculated by summing the scored answers of each item. The resulting total scores were subsequently categorized into quartiles. Mothers with a total stress score below the 25^th^ quartile were defined as lowly, above the 75^th^ quartile as highly stressed. The second and third quartiles were summarized as medium stress. To validate this categorization the concentration of the stress marker homovanillic acid normalized to creatinine^19^,^20^ was determined in the maternal urine at 36^th^ week of gestation as previously described^21^.

### Whole Genome Bisulfite Sequencing

Whole blood samples of 5 mother-child pairs with a low stress score compared to 5 pairs with a high stress score were evaluated. Bisulfite converted DNA from cord blood and venous blood of mothers obtained at the 36^th^ week of gestation were subjected to whole genome bisulfite sequencing (WGBS, Table E2). Using the bsseq v0.10 package for R statistical software v3.0.0 we followed a slightly adapted approach^22^ as described by Hansen *et al*.^23^ to identify regions of differential DNA methylation (DMRs) since they have been reported to be more informative for a phenotype^24^,^25^ compared to single differentially methylated CpGs (Supplementary Information).

### Validation analysis: MassARRAY and qPCR

Methylation differences of selected differentially methylated regions (DMRs) were assessed using the MassARRAY system (Sequenom Inc./Agena Bioscience GmbH, Hamburg Germany), a mass spectrometry based approach for targeted methylation analysis^26^. Since DNA samples were not available for all children, the methylation analyses were restricted to 324 and 217 children at time of birth and at year four, respectively (Figure E1, Table E3). Differential transcription was assessed using the 96.96 Dynamic Array integrated fluidic circuits (Fluidigm, San Francisco, CA, USA).

### Statistics

Equal parameter distribution in our sub- and the entire LINA cohort was tested using Chi-square. To assess whether maternal stress, or DNA methylation and gene expression changes observed in our subcohort contribute to an increased risk for the child to develop persistent wheeze logistic regression models were implemented adjusting for known confounding factors of lung disease in early childhood^27^ (gender of the child, siblings, smoking during pregnancy, ETS exposure after birth, cat keeping, parental history of atopy and parental educational level).

After logarithmic transformation of DNA methylation and gene expression values adjusted mean ratios (MR), i.e. the ratio of the geometric mean, were calculated to evaluate their relationship to the maternal stress level. Confounding factors used were: gender of the child, smoking during pregnancy, ETS exposure after birth (where applicable), age of the mother at time of birth, birth week, mode of delivery, maternal medication during pregnancy^28^ and cell composition (Supplementary Information). All calculations were performed in STATISTICA for Windows, Version 10 (Statsoft Inc., Tulsa, OK, USA). P-values < 0.05 were considered significant.

## Results

### Prenatal maternal stress and persistent childhood wheeze

The median total stress score of mothers in our subcohort was 1.85 (min = 1.00, max = 3.55). Total stress score grouping into quartiles resulted in n = 110, 221 and 112 mothers with a low, medium and high stress level respectively (Table 1).

**Figure 1:**
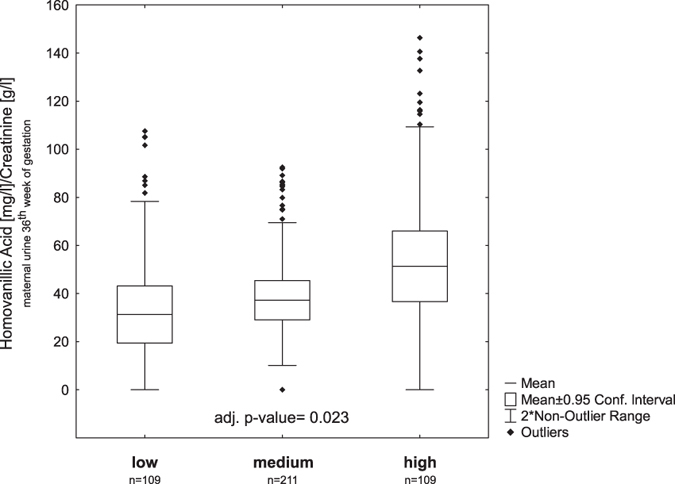
Concentration of homovanillic acid for different stress scores. Homovanillic acid was measured in urine of mothers at 36^th^ weeks of gestation and normalized to creatinine^19^,^20^,^21^. The concentration of the stress metabolite homovanillic acid is significantly higher in mothers with a high stress score compared to those with the lowest stress score supporting stress categorization based on our questionnaire. A significant relationship of homovanillic acid concentration and maternal stress score was determined by multiple regression analysis. Mean ratios were adjusted for maternal medication information derived from the same samples^28^ (box plots represent: mean +/− CI, whiskers +/− non-outlier range).

Urinary homovanillic acid concentration was significantly elevated in mothers with high compared to mothers reporting low stress. This effect sustained even after adjusting for maternal medication a possible influencing factor of homovanillic acid production (adj. p-value = 0.023, Fig. 1). High levels of maternal stress during pregnancy were associated with an increased risk of persistent childhood wheeze (OR = 2.44; 95% CI: 1.09–5.46). Adjustment for possible confounders^27^ slightly increased the calculated OR (OR = 2.73; 95% CI: 1.13–6.55, Table 2).

### Stress and DNA methylation changes

Comparison of five mother-child pairs with low and five pairs with high stress levels by WGBS revealed a substantial genome-wide perturbation in DNA methylation (Table E4). A total of 2306 stress-dependent differentially methylated regions (DMRs, ≥3 consecutive CpGs, Δmeth >10%, t > +/− 4.5^23^) were identified in children and 2495 in mothers with a false discovery rate of 1.9% and 2.5% respectively (Supplementary Information). It is noteworthy that the majority of the DNA methylation changes we detected would not have been observed using a 450 K bead array approach, since only about 5% of DMRs overlapped with at least one CpG sites covered by the array (mothers: n = 130 (5.2%); children: n = 114 (4.9%), Table E4).

**Figure 2:**
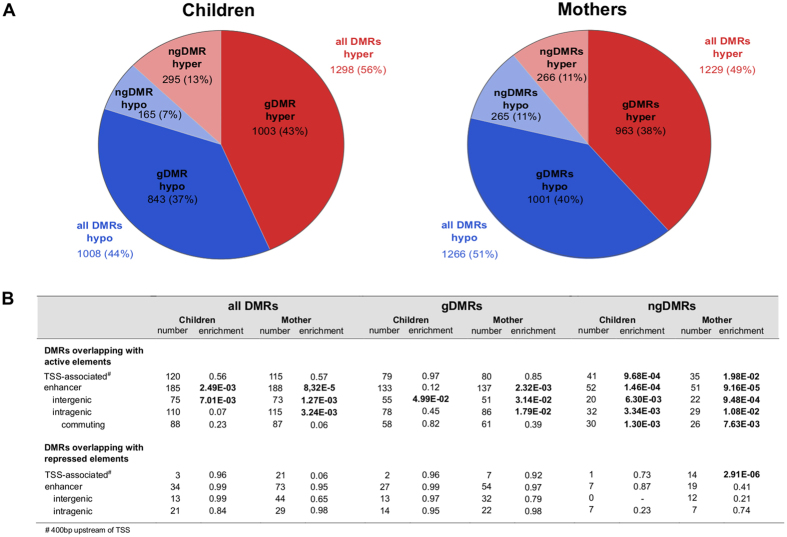
Global overview of differential methylation in children and mothers. (**A**) Pie charts represent the number and percentage of DMRs observed in mothers and children. Two classes of DMRs were differentiated: gDMRs for which methylation significantly correlated with a meQTL-SNP and ngDMRs where the underlying sequence variation did not influence methylation (red: hypermethylation, blue: hypomethylation). (**B**) The table summarizes the functional genomic regions enriched for an overlap with these DMRs. Active regulatory genomic regions in particular enhancer elements were significantly enriched in overlapping DMRs irrespective of their type (gDMR or ngDMR).

DNA methylation cannot only be affected by environmental factors but is also related to the individual genotype. Besides single nucleotide polymorphisms (SNP) that may destroy the CpG context and thereby directly induce differential DNA methylation, SNPs may also create or disrupt transcription factor binding sites (TFBS) affecting the level of DNA methylation^29^. To take this into account we performed SNP-calling for each individual bisulfite-sequencing sample using Bis-SNPs^30^ followed by DMR classification. Whenever a SNP in the neighborhood (+/− 5 kb) of a DMR was statistically significantly correlated with methylation of this DMR, this SNP was classified as a meQTL-SNP (methylation quantitative trait locus) for this DMR. The corresponding potentially genetically influenced DMR was abbreviated as gDMR. All DMRs that were not associated with any meQTL-SNP in their neighborhood and thus presumably not influenced by the genotype were categorized as non-genetically influenced DMRs (ngDMR). This conservative classification still retained about 20% (460/2306 in children and 531/2495 in mothers) of all DMRs as ngDMRs that do not have an apparent correlation with the underlying genetic sequence variation (Fig. 2A).

The total number of DMRs was similar in mothers and children. In children the number of hypermethylated DMRs was significantly higher than in mothers (Chi-square, p < 0.001, power = 1.0, Fig. 2A/B). Only about 8% of DMRs were shared between mothers and children (n = 187, Table E3). The genomic distribution was similar in mothers and children with approximately half of the detected DMRs located intergenically (1286 (52%) and 1256 (55%) in mothers and children respectively, Table E4). Of the remaining DMRs almost all were found in introns (mothers: 44%, children: 41%, Table E4).

DMRs-irrespective of their type (gDMRs or ngDMRs)-preferentially overlapped with functional regulatory regions (Fig. 2B, Table E5). ChIP-Seq data of several histone modifications in a selected set of mothers and children of the LINA cohort^31^ revealed that in particular ngDMRs were enriched in TSS-associated (400 bp upstream of TSS) active regulatory elements and enhancers for both mothers and children (Fig. 2B). The majority of enhancers overlapping with a ngDMR were intragenic (children: 110/185, mothers: 115/188), with more than 75% of these enhancers interacting not only with their host gene but also with distal genes (commuting enhancer, Fig. 2B).

Analysis of DMR overlap with tissue/-cell type specific enhancer regions of the ENCODE roadmap^32^ revealed an anticipated strong enrichment in blood cell specific enhancers (Figure E2, Supplementary Information). This enrichment was particularly pronounced in ngDMRS while gDMRs were also found in enhancer regions characteristic also for other cell types.

**Figure 3:**
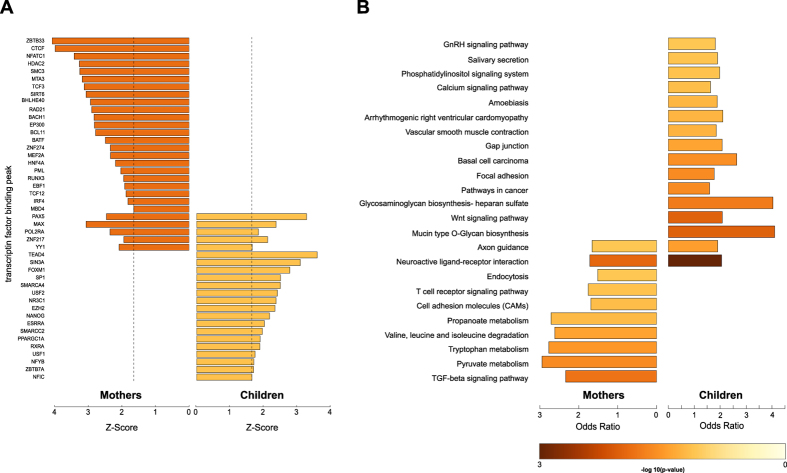
DMR overlap with transcription factor binding sites and KEGG pathways. (**A**) Z-scores of DMR overlaps with transcription factor binding peak sets from ENCODE. For each transcription factor, z-scores were calculated using the mean value and the standard deviation from the overlap distribution of randomly shuffled DMRs with the corresponding transcription factor binding factor binding peak set. The dashed line represents the 5% upper tail of the standard-normal distribution. Only z-scores are shown that are above the 10% upper tail threshold. (**B**) Enrichment of DMRs in KEGG pathways.

In a subsequent analysis we evaluated the overlap of DMRs with TFBS (Fig. 3A, Supplementary Information). Using ENCODE ChIP-Seq data of transcription factor binding^32^,^33^,^34^ we determined a significant overlap of DMRs with POL2RA binding sites in mothers and children supporting our observation that DNA methylation changes not occur arbitrary but particularly affect functional regulatory regions.

In addition, DNA binding sites of the glucocorticoid receptor (*NR3C1*), the main regulator of the glucocorticoid (cortisol in humans) mediated stress response, were significantly enriched for an overlap with DMRs in children (Fig. 3A).

### Minimal functional overlap between mothers and children

DMRs of mothers and children were subjected to an enrichment analysis for KEGG pathways. There was only marginal overlap between enriched pathways while the majority was unique to mothers or their children (Fig. 3B). Most of the receptors in the “Neuroactive ligand receptor interaction” list affected by differential methylation are G protein coupled receptors (GPCR, Fig. 3B) of the neuroendocrine system. Although the number of DMRs in this list was almost identical in mothers and children only eleven genes were consistently affected by DMRs (Figure E3, Table E5A) and of these only five DMRs were shared (Table E5B).

**Figure 4:**
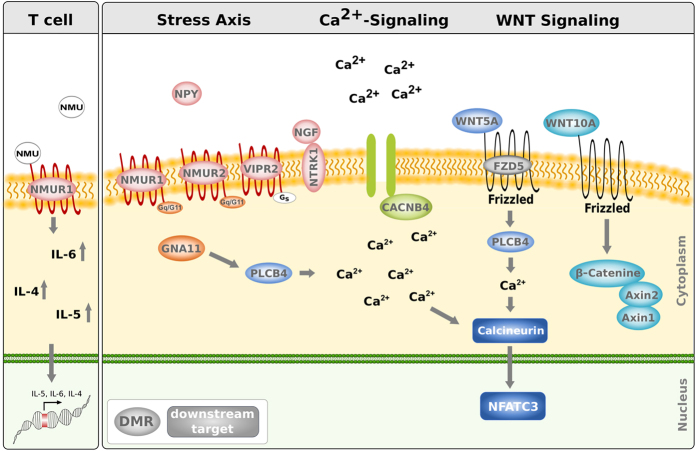
Pathways intertwined by stress induced differential methylation.

A subclass of neuroactive GPCRs promotes calcium release upon activation and is directly linked to the “Calcium signaling pathway” (Figure E4A) enriched only in children for stress-induced DMRs (Fig. 3B). Furthermore, the “Wnt signaling pathway” another pathway mediating intracellular calcium release (Figure E4B) was enriched in DMRs observed in children of highly stressed mothers. We were particularly interested in changes of the epigenetic landscape, which might contribute to the adverse respiratory outcome observed in children. Since calcium homeostasis and Wnt signaling have previously been described as being important for lung development and showed a close interconnection also to the upstream GPCRs mediating stress response we chose candidate genes in the overlap of these pathways (Fig. 4)^35^,^36^. We specifically selected DMRs located in regulatory genomic elements since such perturbations in DNA methylation are more likely to translate into transcriptional changes.

### *NMUR1*, neuroendocrine system and immune response

**Figure 5:**
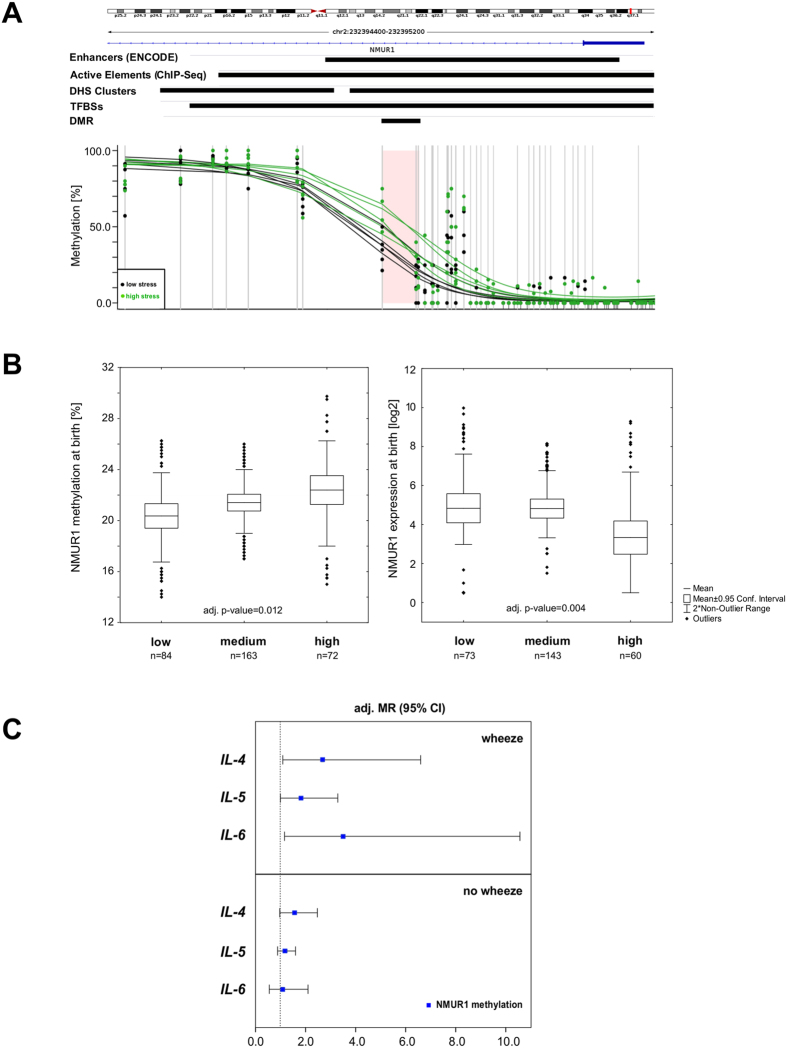
Neuromedin U Receptor 1 is differentially methylated and expressed in children experiencing prenatal maternal stress. (**A**) Based on the histone modification data the DMR (WGBS: Δmeth = 28%) identified in the *NMUR1* gene is located in an enhancer region (ENCODE/Active Elements (own ChIP-Seq data)). (**B**) Validation of the *NMUR1* DMR (7 CpGs, chr2: 232,394,701–232,394,805) in the total cohort by MassARRAY shows that an increase of the maternal stress level leads to a significant elevation in the methylation level in children at time of birth. In highly stressed children this methylation increase corresponds to a significantly decreased mRNA expression of *NMUR1* (mean +/− 95% CI, whiskers +/− non-outlier range). Relationship between *NMUR1* methylation and maternal stress score were determined by multiple regression analysis adjusted for gender of the child, birth week, age of the mother, mode of delivery, maternal smoking/-medication during pregnancy, parental history of atopy and cell composition. (**C**) Relationship of interleukin-4, -5 and -6 secretion at time of birth and *NMUR1* methylation. Data are presented as ratios of the mean (MR) and 95% confidence intervals. Models were adjusted for known confounders of interleukin concentrations in cord blood (month of birth, mode of delivery, gender of the child, parental history of atopy, smoking during pregnancy and cell composition).

In children, we determined a hypermethylated ngDMR in the gene of neuromedin U receptor 1 (*NMUR1*), a GPCR known to be involved in hypothalamic-pituitary-adrenal axis (HPA) response^37^,^38^. The DMR (WGBS: Δmeth = 28%, p-value = 0.030) consisted of three CpGs located in the first intron and overlapped with an ENCODE enhancer region (Fig. 5A). For validation analysis in the entire cohort by MassARRAY, we extended the region to include seven subsequent CpGs. At time of birth methylation in the *NMUR1* DMR significantly increased dependent on the maternal stress score (Δmeth = 2%, adj. p-value = 0.012), although with a much smaller effect size than observed by WGBS. Comparing methylation levels in the same children obtained by WGBS and MassARRAY respectively reveals that this is most likely related to a much smaller dynamic range of the validation method in this region (Figure E5). Correspondingly, a significant decrease in the expression of *NMUR1* in children born to highly stressed compared to those born to lowly stressed mothers was observed (adj. p-value = 0.0034), Fig. 5B).

Since activation of NMUR1 by its ligand neuromedin U is known to elicit Th2-cytokine release^39^, we further investigated the relationship of *NMUR1* methylation and the concentration of IL-4, IL-5 and IL-6 measured in maternal blood four weeks before birth and cord blood. While no stress-dependent changes in IL-4, IL-5, and IL-6 were observed in mothers, children exposed to high maternal stress during pregnancy showed a significantly higher concentration in cord blood of all three cytokines than children exposed to low maternal stress (Figure E6). Applying linear regression models adjusted for possible confounders of interleukin concentrations observed in cord blood revealed a significant relationship between *NMUR1* methylation and IL-4, and IL-6 concentrations at time of birth only in those children developing late or persistent wheeze later in their life’s (Fig. 5C).

Together with the perturbed protein secretion of IL-4, IL-5, and IL-6 the differential methylation in the *NMUR1* enhancer region had subsided in four-year-old children (Figure E7).

### Perturbed calcium signaling and wheezing

In total 19 DMRs in children were assigned to the “Calcium signaling pathway”. One of these DMRs was located in an intron of the *GNA11* gene coding for the alpha subunit of the G protein of the G11 class. *GNA11* is assigned to the KEGG “Calcium signaling pathway” linking neuroendocrine/-transmitter signaling to calcium release (Figure E4A). The *GNA11* gDMR contained eight subsequent CpGs in a poised enhancer region (containing the active mark H3K4me1, Fig. 5A)^31^. Based on ENCODE ChIA-PET data this differentially methylated enhancer interacts with the promoter of *GNA11* (Fig. 5A).

**Figure 6:**
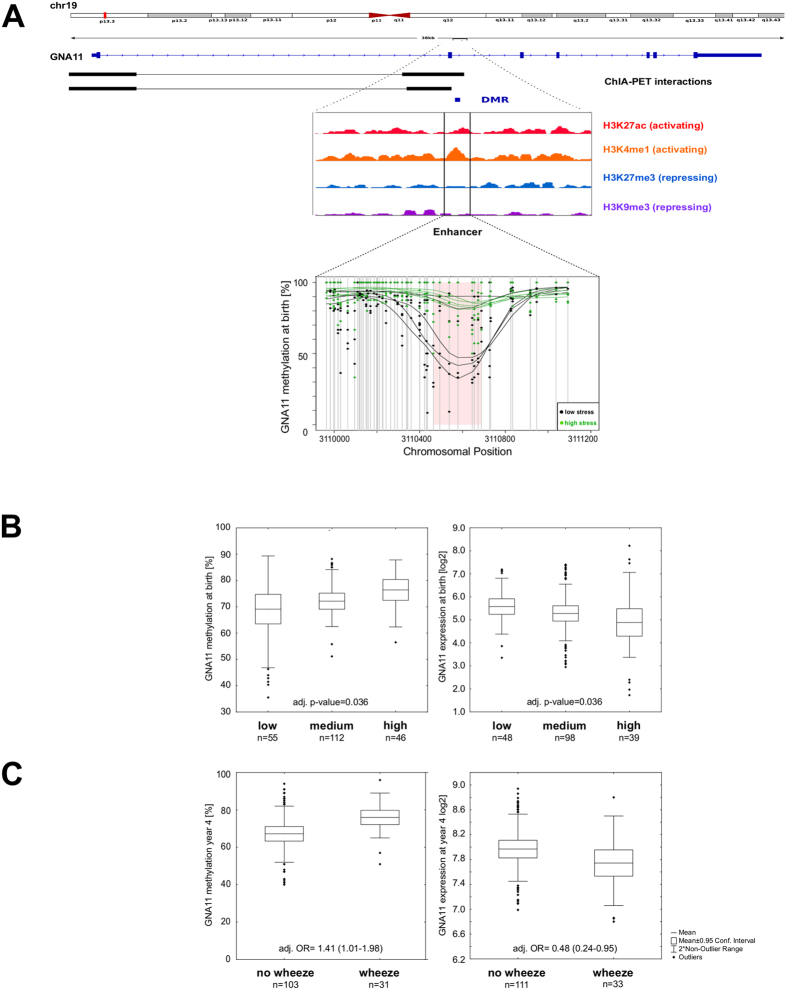
G protein subunit alpha 11 is epigenetically perturbed in children by prenatal maternal stress. (**A**) Although found in an intron the DMR (WGBS: Δmeth = 22%) in the *GNA11* gene is located in an enhancer region according to our own ChIP-Seq data. ChIA-PET data suggest this enhancer regulates its host gene. (**B**) Maternal stress and the methylation level in the *GNA11* DMR show a significant relationship at time of birth while at the same time a significant reverse relationship is observed for the expression of *GNA11* when including all children in our cohort for whom persistent or never persistent wheeze was reported (mean +/− 95% CI, whiskers +/− non-outlier range, p-values adjusted for: gender of the child, birth week, age of the mother, mode of delivery, maternal smoking/-medication during pregnancy, parental history of atopy and cell composition). (**C**) In four-year-old children increased methylation in the *GNA11* enhancer (chr19: 3110675) is associated with an increased risk for persistent wheeze. Corresponding results are observed for *GNA11* expression. Odds ratios were calculated using logistic regression models adjusted for known confounders of wheezing (parental history of atopy, parental educational level, gender, siblings, smoking during pregnancy, ETS exposure after birth, cat keeping and maternal stress score).

Mean methylation of this *GNA11* enhancer was significantly greater in children of mothers reporting high compared to those reporting low stress (WGBS: Δmeth = 22%, p-value = 0.042) (Fig. 6A). High levels of prenatal stress were associated with a significant higher methylation level in this region with a concomitant decrease in gene expression (Fig. 6B). Among persistently wheezing children assessed at age 4, we observed a significantly higher methylation level in the *GNA11* gDMR (adj. OR = 1.41 (1.01–1.98)) with a concomitant lower level of *GNA11* gene expression (adj. OR = 0.48 (0.24–0.95)) when compared to non-symptomatic children (Fig. 6C).

**Figure 7:**
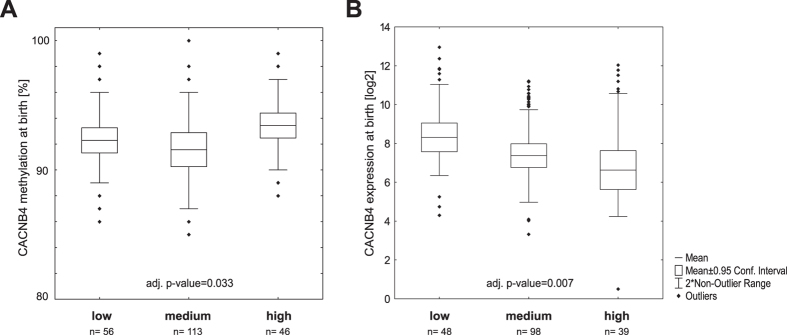
Peripheral beta 4 subunit of voltage gated calcium channel in children is altered by maternal stress. (**A**) Methylation could only be assessed for one CpG in the *CACNB4* ngDMR. Amplification of the entire region yielded no PCR product therefore we could only consider two CpGs (chr2: 152898920–152899112). Since one of the two CpGs was a CpG destroying SNP further analysis was restricted to a single position. (**A**) At time of birth methylation of this CpG shows a significant relationship to the maternal stress score (considering all children either persistently wheezing or showing no respiratory symptoms), (**B**) while in the same children expression of *CACNB4* is decreased to maternal stress. Adjusted p-values were calculated including gender of the child, birth week, age of the mother, mode of delivery, maternal smoking/-medication during pregnancy, parental history of atopy and cell composition as confounding variables.

Among the 18 additional DMRs associated to “Calcium signaling pathway”, several are related to calcium channels including *CACNB4*. The hypermethylated ngDMR located in a weak enhancer region in the *CACNB4* gene likely interacts with its host gene promoter based on ENCODE ChIA-PET data (Table E3). Multiple regression analysis revealed a significant relationship of *CACNB4* methylation and maternal stress score at time of birth (adj. p-value = 0.033, Fig. 6A) with an increase in methylation concomitant with the stress score. At the same time transcription of *CACNB4* was significantly decreased (adj. p-value = 0.007, Fig. 7B).

### Wnt/Ca2+ -signaling pathway and stress-related wheeze

**Figure 8:**
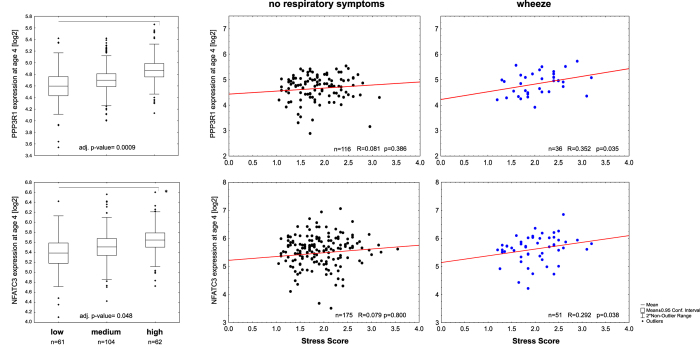
Downstream targets of the Wnt5A/Ca2+ -signaling pathway are activated in prenatally stressed children. mRNA expression in prenatally highly stressed children is significantly increased for the calcium binding beta subunit of calcineurin *PPP3R1* and its downstream target *NFATC3* in four year old children (given are p-values adjusted for: gender of the child, birth week, age of the mother, mode of delivery, maternal smoking/-medication during pregnancy and parental history of atopy; +/− 95% CI, whiskers +/− non-outlier range). A positive correlation of the stress score with *NFATC3* expression was only observed in late and persistently wheezing children, no correlation of maternal stress score and transcription was observed in children without any respiratory symptoms for both PPP3R1 and NFATC3.

We identified several DMRs related to members of the canonical and calcium-dependent Wnt-signaling pathway (Table E6A) To elucidate the functional impact of these DMRs, we determined transcriptional changes of their downstream targets. For the Wnt/Ca2+ -signaling pathway we evaluated transcription of *PPP3R1/PPP3CA* (calcineurin) and *NFATC3*. While no differential transcription was observed for *PPP3CA* (data not shown), *PPP3R1* was significantly elevated in four-year-old children dependent on maternal stress (adj. p-value = 0.0009, Fig. 8). Expression was positively correlated with the stress score only in those children who persistently wheezed. In children of highly stressed mothers, *NFATC3* transcription significantly increased dependent on the maternal stress score in four-year-old children (adj. p-value = 0.048). A positive correlation of the stress score with *NFATC3* expression was only observed in late and persistently wheezing children. For both *PPP3R1* and *NFATC3* no correlation of maternal stress score and transcription was observed in children without any respiratory symptoms (Fig. 8).

While *CTNNB1* (beta-catenin) showed a stress-dependent increase of transcription already at time of birth (Figure E8A, adj. p-value = 0.024), in four-year-old children expression of both *CTNNB1* and *AXIN2* (canonical Wnt-signaling) were significantly elevated in children of highly stressed mothers (Figure E8A/B, adj. p-value = 0.002, 0.004) compared to those of low stressed mothers. Furthermore, elevated *CTNNB1* expression levels at time of birth were associated with an increased risk for the child to develop late or persistent wheeze later in life (adj. OR = 1.78 (1.28–2.89)). At year four differential transcription related to late or persistent wheeze was no longer observed neither for *CTNNB1* nor for *AXIN2* (Figure E8A/B).

## Discussion

Maternal distress during pregnancy has been shown to persistently affect the health of the child in different ways. Premature birth, low birth weight^40^ and an increased risk for childhood adiposity^41^ have been attributed to prenatal maternal stress. Well studied are the long-term neurobiological or behavioral consequences for the child including the development of autism^42^,^43^, depression, or schizophrenia^44^. While these pathophysiological changes in the offspring have been mainly ascribed to reprogramming of neurodevelopment and function of the HPA axis^45^,^46^,^47^ or metabolism on the cellular level^48^ little is known about the pathways contributing to the development of adverse respiratory outcomes like wheezing^1^,^49^, or asthma^50^,^51^,^52^.

Given that the prenatal period is susceptible to external stimuli that can shape the epigenetic landscape and thereby determine disease susceptibility later in life the aim of the current study was to evaluate global DNA methylation changes relating prenatal maternal stress to the increased risk for the child to develop wheezing.

### Global methylation changes

We studied differential DNA methylation at time of birth by WGBS of 10 mother-child-pairs complemented by longitudinal targeted methylation and transcriptional analysis in over 300 children. Our WGBS approach offered us the advantage of a global evaluation of DNA methylation changes not restricted to a selected set of CpGs as in commonly used DNA methylation arrays. This is noteworthy since only 5% of our identified DMRs showed an overlap with CpG sites covered by the Infinium HumanMethylation450 BeadChip array, which would have left the vast majority of the maternal-stress-dependent DMRs undiscovered.

Both mothers and children showed genome-wide perturbations in DNA-methylation affecting genomic regulatory elements in particular enhancer elements. Although this preferential deregulation in DNA methylation has already been described in disease states especially in cancer^53^,^54^, our results show that also changes in the prenatal environment can lead to perturbed enhancer methylation already at a time at which no disease phenotype has yet developed. Epigenetic perturbations in DNA methylation by stress are not random but rather preferentially occur in enhancer elements regulating more than one gene in the genome, which might contribute to the broad ramifications for children’s health attributed to maternal prenatal stress.

A variety of different biological pathways showed an enrichment of DMRs with a minimal overlap between mothers and children. DNA methylation is thought to be subjected to age-dependent changes^55^,^56^ therefore it is possible that the same stressor might lead to distinct perturbations in DNA methylation in different age groups. This is in line with our observation that only genes related to axon guidance and neuroendocrine/neurotransmitter receptor interactions were affected both in mothers and their children with a marginal overlap of affected regions. Since axon guidance is fundamental to neuron wiring modification of the epigenetic signature of this pathway-in particular during brain development^57^-might influence plasticity of the brain and thereby contribute to the adverse psychological conditions described above for maternal stress exposed offspring.

Stressful life events have been widely related to changes in the cortisol mediated stress response^58^. DNA methylation of *NR3C1* (“glucocorticoid receptor”) as the key mediator of this response has been associated with different types of stressful life events. However, the extent and direction of the alteration in DNA methylation vary dependent on the kind of stress experienced^59^,^60^,^61^ and significant differential DNA methylation seems to be observed only in cases of severe psychological trauma^62^,^63^. Previous studies in cord blood showed association of DNA methylation in the 1F region of *NR3C1* with maternal depression or pregnancy related anxiety however no differential methylation was observed related to maternal stress assessment^64^,^65^. Correspondingly our approach based on the identification of differential DNA methylation did not reveal a *NR3C1* related DMR. However, we found a significant enrichment of DMRs overlapping with binding sites of *NR3C1*. This suggests that perturbations in cortisol mediated signaling might also occur independent of a direct effect of maternal stress on *NR3C1* methylation in the child.

### Perturbed pathways are intertwined

It has been accepted that perturbations in the mammalian stress response contribute to an aberrant immune response and may adversely affect lung development in prenatally exposed children^13^,^66^. We therefore focused our validation analysis on DMRs relating to genes connecting the stress response to downstream pathways possibly involved in immune regulation and lung development or function (Fig. 4).

Calcineurin/NFAT signaling is activated by GPCR-mediated intracellular calcium increase. In knockout mice it has been shown that calcineurin/NFAT signaling controls lung maturation prior to birth and deletion of the calcium binding subunit calcineurin b (*PPP3R1*) results in immaturity of the lung^67^. We observed hypermethylation and transcriptional repression for a variety of calcineurin/NFAT upstream regulators related to prenatal maternal stress suggesting an impaired activation of calcineurin/NFAT already during the prenatal period.

The GPCRs *NMUR1* and *-2* play a role in central and peripheral stress response. Binding of NMU to its receptors mediates intracellular calcium signaling^68^, which in T cells promotes synthesis and release of the in allergic inflammation involved Th2 cytokines IL-4, 5, and 6^39^. Our observations linking maternal stress, *NMUR1* methylation, and elevated IL-4, -5, and IL-6 cord blood levels support these findings.

The hypermethylated DMR in an enhancer of NMUR1 we identified in children of highly stressed mothers was confirmed in the entire LINA cohort. Although the observed methylation difference in the larger sample subset was rather small, this seems to be related to a much narrower distribution of methylation values observed by MassARRAY compared to WGBS. The around 5% higher methylation found in prenatally stressed children might be related to a deregulated subset of T cells producing Th2 cytokines. *NMUR1* enhancer hypermethylation was accompanied by repressed transcription of *NMUR1* in prenatally stressed children. This result is in accordance with data suggesting a decrease in *NMUR1* expression in response to stress^69^,^70^. A negative feedback in stress activated receptor signaling is common^71^. Therefore it seems likely that hyperactivation of NMU mediated stress response perturbed *NMUR1* methylation and transcription. When exposure to maternal stress is ceased, the methylation and transcription changes of *NMUR1* subside together with the altered immune response (elevated IL-4, -5, -6) observed at birth.

Activation of NMUR1/2, coupled to G alpha q/11 (*GNA11*) and G alpha i^72^, can lead to activation of phospholipase C mediated increase in cytoplasmic calcium. Both *GNA11* and *PLCB4 “phospholipase C beta 4”* showed a hypermethylated enhancer regions related to prenatal maternal stress. For *GNA11* this was associated with decreased expression in both prenatally stressed and children with wheezing symptoms. Similar observations for the calcium channel coding *CACNB4* gene further suggest an involvement of calcium signaling in impaired lung function.

Wnt-signaling plays a widely established role in lung organogenesis. In particular canonical Wnt-signaling is involved in cell fate decisions and differentiation of lung cells^73^. Although the role of non-canonical/WNT5A signaling upstream of calcineurin/NFAT in lung development is less clear, *Wnt5a* knockout mice are characterized by morphologically smaller lungs, thickened mesenchyme and a delayed alveolar development^74^,^75^. Albeit a variety of DMRs related to members of both Wnt-signaling pathways observed, our results point to calcium-dependent Wnt-signaling as related to the development of wheezing symptoms. We did not observe long-term epigenetic memory in the investigated regions, which were mostly ngDMRs. However epigenetic modifications upstream of calcineurin/NFAT signaling in the prenatal period might facilitate altered lung development contributing to the wheezing phenotype observed in children exposed to high levels of prenatal maternal stress.

Our approach revealed extensive alterations in the epigenome of mothers and children preferentially in enhancer region, which might contribute to the broad ramifications attributed to stress exposure. Although we report blood-derived DNA methylation changes, which may not always reflect methylation within other tissues.

Based on WGBS data from 10 mother-child pairs we derived a hypothesis how maternal stress in the prenatal period might be translated into impaired lung function in the child. We identified potentially involved signaling pathways and targets, which were subsequently validated in a larger sample set from the entire LINA cohort using targeted approaches. Since the focus of the present study was on prenatal stress and respiratory outcomes, pathways and targets involved in stress signaling and lung development were selected for further validation analyses thereby ignoring stress-related epigenetic modifications in pathways potentially linked to further outcomes, such as overweight development or adverse psychological conditions. Addressing all adverse outcomes described to occur in children exposed to maternal prenatal stress was beyond the scope of this study but certainly deserves further in depth analysis in follow-up studies.

## Additional Information

**How to cite this article**: Trump, S. *et al*. Prenatal maternal stress and wheeze in children: novel insights into epigenetic regulation. *Sci. Rep.* **6**, 28616; doi: 10.1038/srep28616 (2016).

## Supplementary Material

Supplementary Information

Supplementary Information

## Figures and Tables

**Table 1:** Characteristics of the analysed subcohort and the entire LINA cohort.

Parameters	Discovery cohort	Analysed subcohort	Entire LINA cohort	*P* value*
	**Pregnancy and birth**			
	**n (%), N = 10**	**n (%), N = 443**	**n (%), N = 629**	
**Gender of the child**				
male	7 (70)	227 (51.2)	327 (52.0)	0.858
female	3 (30)	216 (48.8)	302 (48.0)	
**Parental history of atopy**^a^				
negative	5 (50)	149 (33.6)	212 (33.3)	0.913
single positive	4 (40)	213 (48.1)	296 (46.5)	
double positive	1 (10)	81 (18.3)	121 (19.0)	
**Parental education**^b^				
low	0 (0)	5 (1.1)	16 (2.5)	0.297
intermediate	1 (10)	98 (22.1)	144 (22.6)	
high	9 (90)	340 (76.8)	469 (73.7)	
**Pet keeping (cat)**				
no	7 (70)	380 (85.8)	511 (80.3)	0.573
yes	3 (30)	63 (14.2)	95 (14.9)	
**Smoking during pregnancy**				
never	9 (90)	388 (87.6)	534 (89.9)	0.575
occasionally	1 (10)	28 (6.3)	43 (6.8)	
once per week	0 (0)	2 (0.5)	4 (0.76)	
daily	0 (0)	25 (5.6)	48 (7.5)	
**ETS exposure after birth**				
no	10 (10)	422 (95.3)	568 (93.9)	0.326
yes	0 (0)	20 (4.5)	37 (6.1)	
**Maternal stress during pregnancy**				
low	5 (50)	110 (24.8)	151 (24.4)	0.947
medium	0 (0)	221 (49.9)	306 (49.5)	
high	5 (50)	112 (25.3)	161 (26.1)	

^*^P value from chi squared test for cross relationship, analysed subcohort against entire cohort.

^a^history of atopy is defined as: occurrence of asthma or atopic dermatitis or hay fever.

^b^low, 9 yrs of schooling or less “Hauptschulabschluss”; intermediate, 10 yrs of schooling “Mittlere Reife”; high, 12 yrs of schooling or more “(Fach-) hochschulreife”.

**Table 2:** Relationship between maternal stress during pregnancy assessed using the perceived stress questionnaire (PSQ) and children’s persistent wheeze up to the age of 5 years.

**Maternal stress**#	**% (n/N)**	**Persistent Wheeze**	
		**Raw OR (95% CI)**	Adjusted OR (95% CI)*
Low	9.1% (10/110)	1.00	1.00
Medium	12.2% (27/221)	1.39 (0.65–3.00)	1.39 (0.61–3.15)
High	19.6% (22/112)	2.44 (1.09–5.46)	2.73 (1.13–6.55)

^*^Adjusted odds ratios for parental history of atopy, parental educational level, gender of the child, siblings, smoking during pregnancy, ETS exposure after birth, cat keeping.

^#^Maternal stress was assessed by the 20-item PSQ^17^,^18^. A total score was calculated by summing the scored answers of each item. The resulting total scores were subsequently categorized into quartiles. While mothers with a total stress score below the 25^th^ quartile were defined as lowly stressed, those above the 75^th^ quartile were defined as highly stressed.
