# Supplementary material for: Prenatal maternal stress and wheeze in children: novel insights into epigenetic regulation
Source: Sci Rep. 2016 Jun 28;6:28616. doi: 10.1038/srep28616 (PMC4923849; doi:10.1038/srep28616)
Supplement: Supplementary Information [file srep28616-s1.pdf]

Prenatal maternal stress and wheeze in children: novel insights into epigenetic regulation

Saskia Trump, Matthias Bieg, Zuguang Gu, Loreen Thürmann, Tobias Bauer, Mario Bauer, Naveed Ishaque, Stefan Röder, Lei Gu, Gunda Herberth, Christian Lawerenz, Michael Borte, Matthias Schlesner, Christoph Plass, Nicolle Diessl, Markus Eszlinger, Oliver Mücke, Horst-Dietrich Elvers, Dirk K. Wissenbach, Martin von Bergen, Carl Herrmann, Dieter Weichenhan, Rosalind J. Wright, Irina Lehmann, Roland Eils

Supplementary Information

Experimental Procedures

Questionnaire

During pregnancy standardized questionnaires were recorded, collecting data about atopic diseases, educational level, socioeconomic status, and smoking behavior of the parents, housing conditions, pet keeping, and life-style related exposure. Annually, starting at the child's first birthday, disease outcomes of the children were assessed via questionnaire. Wheezing was recorded as a parental report of wheezing symptoms ("whistling or wheezing from the chest,"). Children with late onset wheezing were defined according to Martinez et al.[1] as those who had no wheezing symptoms during the first three years of life but showed wheezing symptoms after this period (at age 4 or 5). Persistent wheeze was defined as the occurrence of wheezing symptoms in the first three years of life with subsequent episodes at age 4 or 5. Never wheezing children did not show any wheezing or other respiratory symptoms up to the age of 5. All questionnaires were self-administered by the parents. During annual clinical visits blood samples were obtained. The study was approved by the institutional review board of the University of Leipzig (046-2006, 160-2008, 160-2008, 160b/2008, 144-10-31052010, 113-11-18042011).

Perceived stress score calculation

The 20-item questionnaire consists of positively and negatively worded questions. To calculate the total perceived stress score negatively worded questions (2, 3, 5, 7, 9, 10, 11, 13, 15, 17, 18, and 20) were scored as (1) hardly ever, (2) sometimes, (3) often, and (4) usually. Positively worded questions (1, 4, 6, 8, 12, 14, 16, and 19), were reversely scored (1) usually, (2) often, (3) sometimes (4) hardly) ever. The total stress score was derived as the mean of all 20 scored questions.

Sample Selection

For WGBS ten mother-child pairs were selected according to the stress score of the mothers. Five mothers with a high level of perceived stress were chosen together with their children and compared to 5 mother child pairs with a low level of maternal stress. The median of the homovanillic acid concentration in the low stressed group was 0 mg/l/g/L (IQR: 0-0), in the high stressed group 75.7mg/l/g/L (IQR:0-103).

The age of stressed mothers (mean=28.57 years, S.D. 3.87) was similar to that of non-stressed mothers (mean=33.56 years, S.D. 5.57, p=0.138 from Student's t-test). The birth weight of the children did not differ significantly between children of stressed and non-stressed mothers (3,000 g vs. 3,340 g, p=0.462 from Student's t-test).

From the 443 children in our sub-cohort 324 and 217 DNA samples were available at birth and year 4, respectively, to investigate differential methylation of the NMUR1 and GNA11 DMRs identified by WGBS. Differential transcription of selected genes was evaluated for 313 children at birth and in 227 children at year 4.

Isolation of gDNA and RNA from whole blood

Genomic DNA from whole blood samples (peripheral blood at 36th week of gestation or cord blood) was isolated using the QIAmp DNA Blood Mini Kit (Qiagen, Hilden, Germany), according to manufacturer's instruction. Total RNA was prepared using peqGold RNA Pure (peqlab, Erlangen, Germany), according to the manufacturer's instruction. RNA integrity was assessed in a subset of samples from our cohort (n=114), the mean RIN value derived was 8.23 +/- 1.63 (SD). The cDNA synthesis was carried out with 5 µg of RNA by using the ImProm-II^{TM} Reverse Transcription System (Promega, Mannheim, Germany).

WGBS library preparation and sequencing

Illumina Libraries were prepared using the TruSeq DNA Sample Prep Kit v2-Set A (Illumina Inc., San Diego, CA, USA) according the manufacturer's instructions. Briefly, 2 µg genomic DNA in 55 µl nuclease-free water (Ambion/Life Technologies GmbH, Darmstadt, Germany) was fragmented using a Covaris S2 ultrasonicator (Covaris, Woburn, Massachusetts, USA) and the following settings: 10% duty cycle, intensity 5, 200 cycles per burst, frequency sweeping, for 6 minutes. The fragmented DNA was end-repaired, extended with an 'A' base on the 3′ end and ligated with TruSeq paired-end indexing adapters. Then, adapter-ligated fragment libraries were treated with bisulfite using the EpiTect Bisulfite Kit (Qiagen) following the instructions in the Illumina WGBS for Methylation Analysis Guide (Part # 15021861 Rev. B). After bisulfite conversion the fragment libraries were directly amplified using KAPA HiFi Uracil+ DNA Polymerase according to the settings for TruSeq™ DNA in the technical Data Sheet (KAPA HiFi HotStart Uracil+ Ready Mix, KR0413 - version 1.12, peqlab, Erlangen, Germany). Two 50 µl PCR reactions per sample were prepared and 14 cycles of PCR performed. Amplified fragment libraries were pooled and purified with 1x Agencourt AMPure XP beads (Beckman Coulter GmbH, Krefeld, Germany). WGBS Illumina Libraries were validated using the 2100 Bioanalyzer (DNA 1000 Kit, Agilent Technologies) and Qubit flourometer (Qubit dsDNA HS Assay Kit, Invitrogen/ Life Technologies GmbH, Darmstadt, Germany).

The final libraries were clustered on the cBot (Illumina Inc.) using TruSeq PE Cluster Kit v3 according the manufacturer`s instructions with a final concentration of either 9 pM or 10 pM (depending on the sample) spiked with 1% PhiX control v3 and an additional dedicated PhiX control lane. Sequencing on HiSeq2000 (101 bp paired-end) was performed using standard Illumina protocols and the 200-cycles TruSeq SBS Kit v3 (Illumina Inc.).

Sequence alignment and cytosine methylation estimation

We used a mapping method as described earlier[2] for reads from conventional whole-genome bisulfite sequencing. Briefly, the hg19 reference genome (37d5) was transformed *in silico* for both the top strand (C to T) and bottom strand (G to A). Before alignment, adaptor sequences were trimmed using SeqPrep (https://github.com/jstjohn/SeqPrep). Then the first read in each read pair was C-to-T converted and the 2nd read in the pair was G-to-A converted. The converted reads were aligned to a combined reference of the transformed top (C to T) and bottom (G to A) strands using BWA (bwa-0.6.2-tpx)[3] with default parameters except the quality threshold for read trimming (-q) of 20 and the Smith-Waterman for the unmapped mate disabled (-s). After alignment, reads were converted back to the original states and reads mapped to the antisense strand of the respective reference were removed. Duplicate reads were removed using (Picard MarkDuplicates, http://picard.sourceforge.net/[4]). Reads with alignment scores less than 1 were filtered before subsequent analysis. Total genome coverage was calculated using the total number of bases aligned from uniquely mapped reads over the total number of mapable bases in the genome.

At each cytosine position, reads that maintain the cytosine status were considered methylated, and the reads that have cytosine converted to thymine were considered unmethylated. Only bases with Phred-scaled quality score of ≥20 were considered.

DMR calling

We followed an approach described by Hansen *et al.*[5] using the bsseq v0.10 package for R statistical software v3.0.0 with adapted window size for smoothing because of sufficiently high CpG coverage. As opposed to at least 70 CpGs, we incorporated at least 10 adjacent CpGs with a minimum window size of 1 kb (h=500), breaking the smoothing if gaps between CpGs exceeded 2 kb (maxGap=2000).

Methylation values of close by CpGs are not independent of each other, so this approach guaranteed very high statistical confidence for a sequence of CpGs and

requires a DMR-model of several consecutively differentially methylated CpGs. Our choice was to define DMRs with at least 3 CpGs as previously described[8]. DMRs were called according to the standard settings recommended in bsseq[5] as ≥3 CpGs with $t \geq |4.5|$, $\Delta_{\beta\text{-values}} \geq 0.1$ and at least one CpG every 300 bp. We included all these CpG position for DMR calling with a coverage of more than 8 reads in at least three individuals of either group (low stress, high stress). An additional stringency filter was added to filter DMRs on the raw data level by calculating average methylation levels of each DMR and sample and performing a moderated t-test (d-statistics, R-package siggenes v1.36.0[6],[7]) to assign *p*-values to each DMR. Based on the *p*-value ($p_{\text{d-statistics}}<0.05$) and the level of mean methylation changes ($\Delta_{\beta\text{-values}} \geq 0.1$ in both raw and smoothed data), we ranked and filtered the DMR list for downstream analyses. Finally, we conducted a permutation analysis to assess a false discovery rate (FDR) of the called DMRs. Sample labels of raw data methylation averages were permuted randomly 100 times with replacement and subsequent calculation of the d-statistics and mean methylation differences ($\Delta_{\text{ß-values}}$). From this, we calculated $\mathrm{FDR}_{median} = \frac{median\ \#\ of\ significant\ in\ permutations}{\#\ of\ significant\ in\ original\ data}$ with the same thresholds of $p_{\text{d-statistics}}$ and $\Delta_{\beta\text{-values}}$ as above.

Pathway enrichment

One-sided Fisher's exact tests identified KEGG pathways (latest public update 2011/03/14) that were significantly over-represented by DMRs. P-values were corrected for multiple testing by the Benjamini-Hochberg procedure (false discovery rate <0.05).

Methylation quantitative trait loci (meQTL) analysis

We first determined the genotypes at known SNP positions from dbSNP (version 141)[9] for all individuals separately using the software Bis-SNP (version 0.78)[10]. Only

SNPs with a Phred-scaled quality score greater or equal to 13 were retained. Afterwards, we performed correlation analysis (Spearman) of mean methylation within DMRs to the genotypes of each SNP within 5kb of the DMR. FDR correction of p-values was done using a randomization approach. First, we randomly shuffled the genotypes at all SNP positions among the individuals, and performed correlation analysis to DMRs for the randomized genotypes. We determined the minimal correlation coefficient ensuring a FDR smaller or equal to 0.1 as follows: Let $P_{real}(r)$ be the probability of any SNP having a correlation coefficient equal to r to a proximal DMR when considering the real genotypes. Let $P_{random}(r)$ be the probability of any SNP having a correlation coefficient equal to r to a proximal DMR when considering the randomly shuffled genotypes. The FDR at a given correlation coefficient r is then defined as

$$fdr(r) = \frac{\sum_{x=r}^{1} P_{random}(x)}{\sum_{x=r}^{1} P_{real}(x)}$$

The minimal correlation coefficient for which a FDR smaller or equal to 0.1 is ensured can be calculated by solving the equation $fdr(r) = 0.1$ with respect to the variable r. This yields a correlation value of 0.7 for both the children, and mothers. Therefore, we defined a DMR as being genetically influenced (gDMR), if there is a SNP in 5kb vicinity showing a correlation coefficient greater or equal to 0.7 to its mean methylation, else we classified the DMR as not being genetically influenced (ngDMR).

Genomic regions enrichment

The enrichment analysis of DMRs within genomic regions of interest (ROIs) was done using a random sampling approach. For a given set of ROIs we randomly shuffled the DMR intervals within a genomic subregion that can potentially contain

DMRs and calculated the number of overlaps of ROI intervals with the DMRs. In concordance with the DMR calling parameters we only used genomic regions for random shuffling that fulfill the following criteria: Each CpG position must be covered by at least 8 reads in at least 3 individuals of each group (low stress, and high stress), and the distance between two neighboring CpGs must be smaller or equal to 300 base pairs. This procedure was repeated 1000 times resulting in an estimate of the overlap distribution of ROI intervals with a random background of DMRs. The mean value μ was then used to fit a Poisson distribution with $\lambda = \mu$ and probability density function $P(\lambda = \mu, x)$. A one sided test for enrichment was performed by calculating a p-value as follows: Let n be the number of overlaps of DMRs with the ROI intervals, then the p-value for enrichment was calculated as

$$p_{val} = \int_{x=n}^{\infty} P(\lambda = \mu, x)$$

The odds ratio was calculated as

$$OR = \frac{n}{\mu}$$

Transcription factor binding sites and enhancer regions

Files containing genomic intervals corresponding to transcription factor binding sites were collected from the ENCODE download section of the UCSC genome browser (http://hgdownload.cse.ucsc.edu/goldenPath/hg19/encodeDCC/wgEncodeRegTfbsClustered/wgEncodeRegTfbsClusteredWithCellsV3.bed.gz)[11,12], containing binding sites for 161 transcription factors generated by ChIP-seq experiments of 91 different cell types.

Enhancer regions were downloaded from the epigenome roadmap homepage (http://egg2.wustl.edu/roadmap/web_portal/chr_state_learning.html#core_15state)[13], and further processed as follows: First we extracted the enhancer state regions (Enh,

and EnhG) from the chromHMM 15 state model files of 126 single cell types/ tissue types over 19 tissue groups. Afterwards, we merged the enhancer regions according to their tissue group. We merged neighboring enhancer regions, if their distance was less than 1kb.

MassARRAY methylation analysis

Quantitative DNA methylation analysis of the *NMUR1*, *GNA11* and *CACNB4* DMR were performed using the Sequenom's MassARRAY platform.

Briefly, genomic DNA from whole blood samples was chemically modified with sodium bisulfite using the EZ methylation kit (Zymo Research, Freiburg, Germany) according to the manufacturer's instructions. PCR primers were designed with an additional T7 promoter tag for *in vitro* transcription for each reverse primer, as well as a 10-mer tag on the forward primer. Bisulfite treated DNA was PCR amplified (*NMUR1* forward primer: aggaagagagTAAGTGAGGGAGTGTTAAGG, *NMUR1* reverse primer: cagtaatacgactcactatagggagaaggctCAAAACRCCTCCCTTAAAC, amplicon coordinates chr2:232394600-232394859; *GNA11* forward primer: aggaagagagTTGTTAGGGTGGGGTTATG, *GNA11* reverse primer: cagtaatacgactcactatagggagaaggctTACCTAATAAACTACTCAACC, amplicon coordinates: chr19:3110404-3110724, *CACNB4* forward primer: aggaagagagGTTTTTGTTTAATAAAGGTTTGG, *CACNB4* reverse primer: cagtaatacgactcactatagggagaaggctACTATCAACAAATAACCACAC, amplicon coordinates chr2:152898920-152899112) using HotStarTaq DNA Polymerase (Qiagen) with the following cycling program: 95°C for 15 min, followed by 45 cycles of 94°C for 30 sec, 52°C for 30 sec, 72°C for 1 min and a final elongation step at 72°C for 5 min on a LightCycler 480 (Roche Applied Science, Mannheim, Germany). The PCR product was in vitro transcribed and cleaved by RNase A using the EpiTyper T Complete Reagent Set (Sequenom Inc./Agena Bioscience GmbH, Hamburg Germany) and subjected to MALDI-TOF mass spectrometry analysis to

determine methylation patterns as previously described[14]. DNA methylation standards (0%, 20%, 40%, 60%, 80%, and 100% methylated genomic DNA) were used to control for potential PCR bias. Expected and measured DNA methylation values were significantly correlated with Spearman correlation coefficients >0.9 for all amplicons used in the validation analysis, supporting the validity of the applied assays (NMUR1: R^2=0.979, GNA11: R^2=0.911, CACNB4: R^2=0.982; Figure E9). As expected all negative control samples (H_2O) included in each MassARRAY assay to control for contaminations or PCR by-products did not derive methylation values.

Adjustment for cellular composition

To account for DNA methylation changes resulting from differences in the cellular composition of our whole blood samples we followed an already suggested strategy[15,16] and determined cell type specific regions of differential methylation to be used as additional confounders in our regression analyses. These data were derived from 450K Illumina Bead arrays available for all children in the investigated subcohort at time of birth.

Briefly, to identify cell type specific marker regions we used publically available methylation data from FACS sorted granulocytes, CD4+ and CD8+ T-cells, NK cells, B-cells and monocytes[15]. Analysis of variance was used to test Beta values of each CpG in every promoter region (1 to 2000nt upstream of TSS) of a particular cell type for statistical significance compared to the other cell populations considered. Post-hoc tests were applied and p-values corrected for multiple testing by Benjamini-Hochberg. Cell type specific marker regions were identified (three consecutive CpGs with significant methylation differences) and ranked by distance of mean methylation in a given cell type to the mean methylation in the next closest cell type. For each cell type a characteristic promoter region was determined to derive the confounding variables to correct for cell type composition of each sample. The regions considered

for each cell type were: granulocytes cg20203469, cg19037107, cg01692842 (*ACAD8*); CD4+ T-cells cg26848442, cg12929678, cg20768743 (*CD226*); CD8+ T-cells cg18830527, cg21648425, cg18174654 (*CD8A*); monocytes cg15309910, cg12037947, cg12873119 (*CD93*); B-cells cg07597976, cg06323049, cg27565966 (*CD19*)[17,18] and NK cells cg15241779, cg09261289, cg13828440 (*KLRD1*).

RNA extraction, cDNA Synthesis, and qPCR

Total RNA was prepared from fresh cord blood by using peqGold RNA Pure (peqlab, Erlangen, Germany) and from in PAXgene Blood RNA Tube collected blood of year 4 by PAXgene Blood RNA Kit (Qiagen), according to the manufacturer's instructions. The cDNA synthesis was carried out with 5 µg of RNA by using ImProm-II™ Reverse Transcription System (Promega, Mannheim, Germany).

Gene expression was measured using the 96.96 Dynamic Array integrated fluidic circuits (Fluidigm, San Francisco, CA, USA) for 313 and 227 children at birth and year four respectively. Intron-spanning primers were designed (Table E6) and UPL probes selected by the Universal Probe Library Assay Design Center (http://qpcr.probefinder.com/organism.jsp). A pre-amplification reaction was performed by pooling all primers (final concentration, 50 nM), 5 µl of cDNA and 2x PreAmp Master Mix (Applied Biosystems/Life Technologies GmbH, Darmstadt, Germany). The cycling program consisted of 95°C for 10 min, followed by 14 cycles of 95°C for 15 sec and 60°C for 4 min on a LightCycler 480 (Roche Applied Science, Mannheim, Germany). The qPCRs of 1:5 diluted with TE buffer preamplified templates were performed following manufacture's instruction for UPL (Roche Applied Science, Mannheim, Germany) assays. Briefly, for each individual assay, a 10X Assay Mix that contained 2 µM of each forward and reverse primer, 1 µM UPL probe and 0.025% Tween-20 was prepared, and 5 µl of the mix was loaded into the assay inlets of the array. Into the sample inlets, 5 µl of the following solution was

dispensed: 2.5 µl of PreAmp sample in 1.1X of FastStart Universal Probe Master Mix (Roche Applied Science, Mannheim, Germany). The cycling program consisted of 2 min at 50°C, 10 min at 95°C, followed by 35 cycles of 95°C for 15 sec, 70°C for 5 sec, and 1 min at 60°C. All reactions were performed in triplicates.

Gene expression values were determined by using the $2^{-\Delta\Delta CT}$ method[19] with *GAPD*, *GUSB*, *PGK1* and *PPIA* as reference genes and normalized to the lowest measured value (for primer sequences see Table E6).

Cytokine measurement

Heparinized blood samples from mother-child pairs were obtained by venipuncture and processed within six hours for further analysis. After incubating for 4 h at 37°C, samples were diluted with RPMI-1640 medium without supplements in a ratio of 1:1 and centrifuged. Cell-free supernatants were collected and stored at -80°C until subsequent analysis. Concentrations of IL-4, IL-5, and IL-6 in the supernatants of whole blood samples were detected by flow cytometry using the BD CBA Human Soluble Flex Set system (BD Bioscience, Heidelberg, Germany) according to the manufacturer's instructions and as described previously[20].

In brief, cytokine specific antibody coated beads were incubated for 1 h with 25 µl of blood samples or standard solution. Thereafter, samples were incubated with the corresponding PE labeled detection antibodies for 2 h. After one washing step samples were measured by flow cytometry. Analysis of data and quantification of cytokines was performed using the FCAP Array™ software (Becton Dickinson, Heidelberg, Germany) on the basis of corresponding standard curves. Finally, plasma dilution factor was accounted.

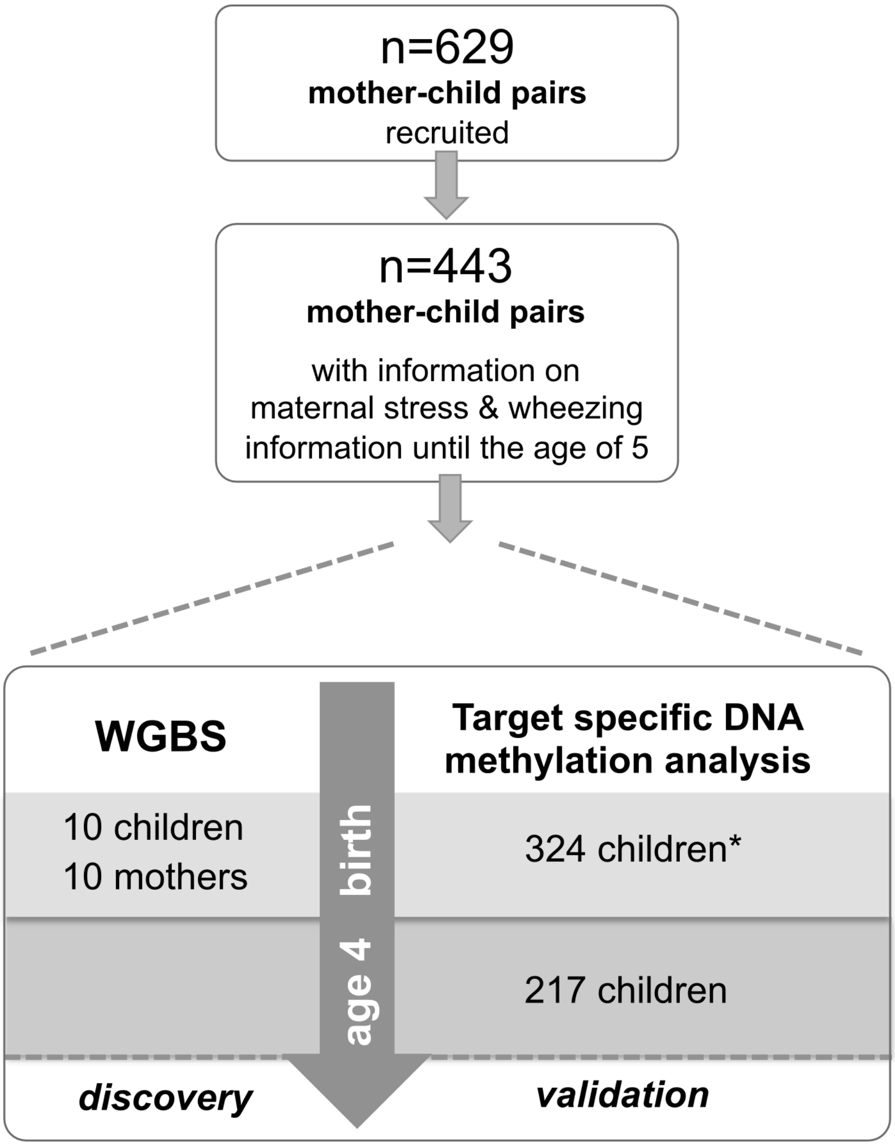

Figure E1. **Overview of DNA samples used in this study.** In the LINA cohort n=629 mother child pairs were recruited and for n=443 pairs a maternal stress score and valid wheezing information for the child until the age of 5 was available. For methylation analysis DNA samples from cord blood (*) of n=324 of those children were available at time of birth. A smaller number of samples (n=217) could be assessed for four-year-old children due to drop out of the study. At both time points investigated no difference between characteristics of the examined subcohort and the entire cohort was observed.

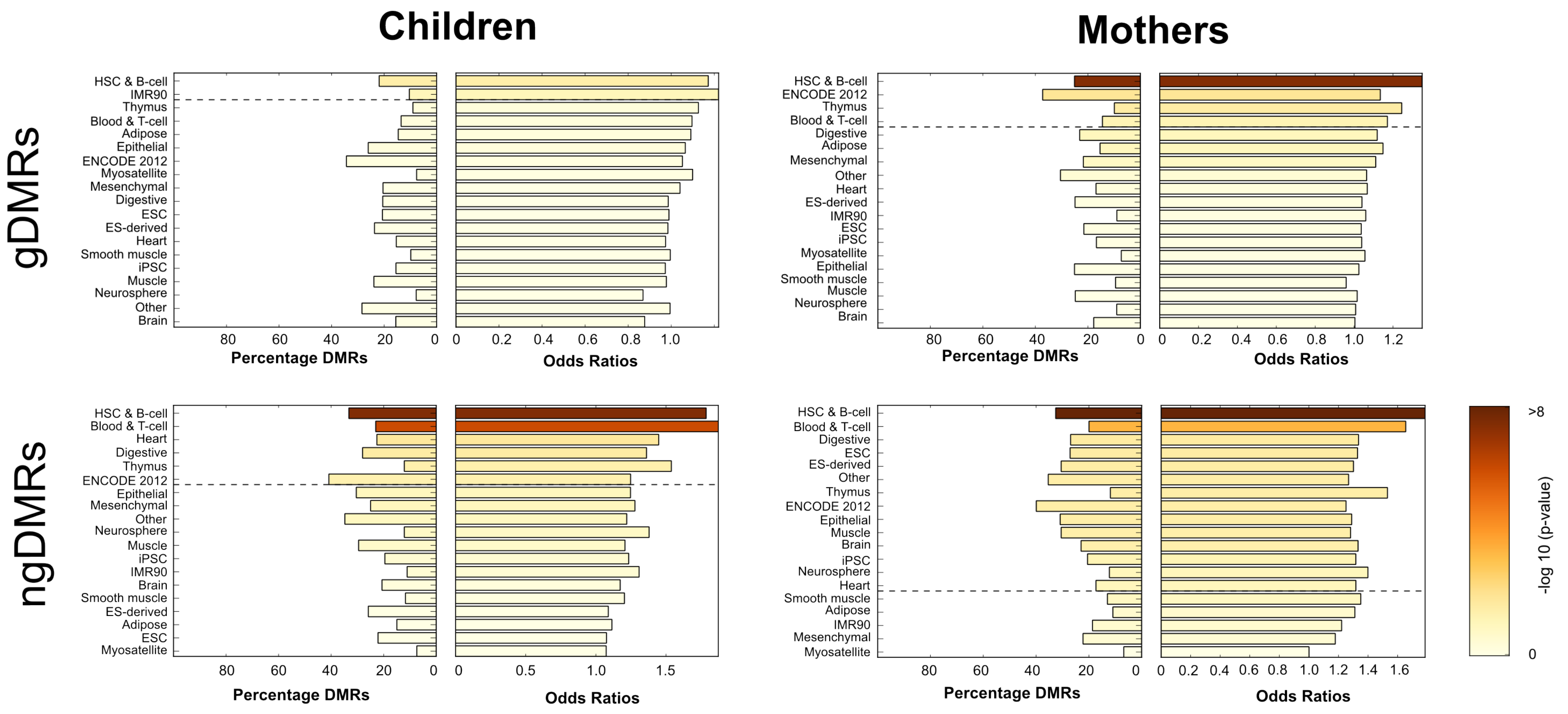

Figure E2. **DMR enrichment in tissue/ cell type specific enhancers.** Nomenclature used for enhancer types according to ENCODE roadmap[13].

A

Children

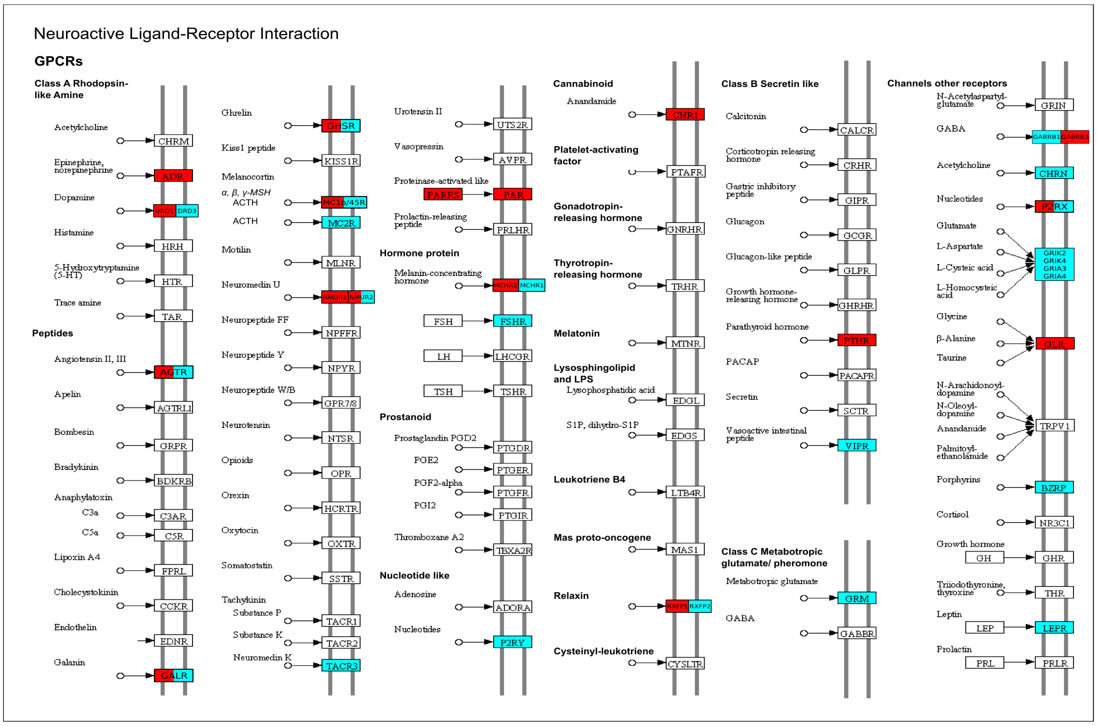

B

Mothers

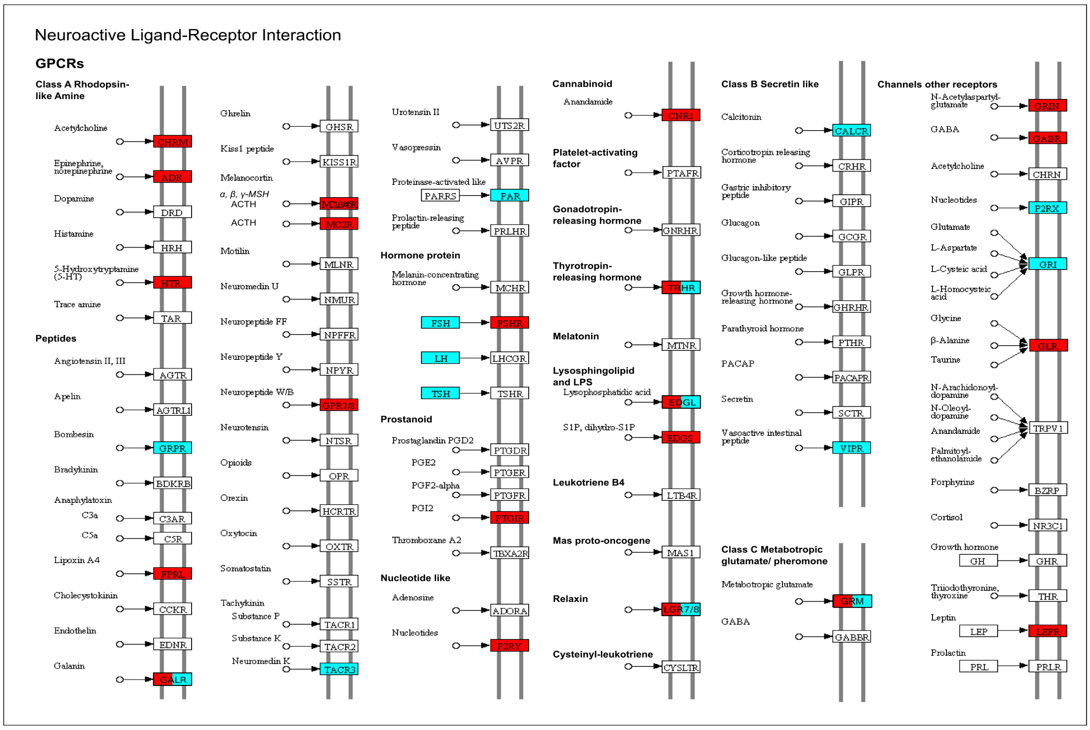

Figure E3. **DMRs in the "Neuroactive Ligand-Receptor Interaction" KEGG-pathway.** This is one of two pathways enriched in DMRs for both children and their mothers. (A) Shows DMRs identified in children, while (B) depicts DMRs of this pathway in mothers. Hypermethylation is shown in red, hypomethylation in blue.

A

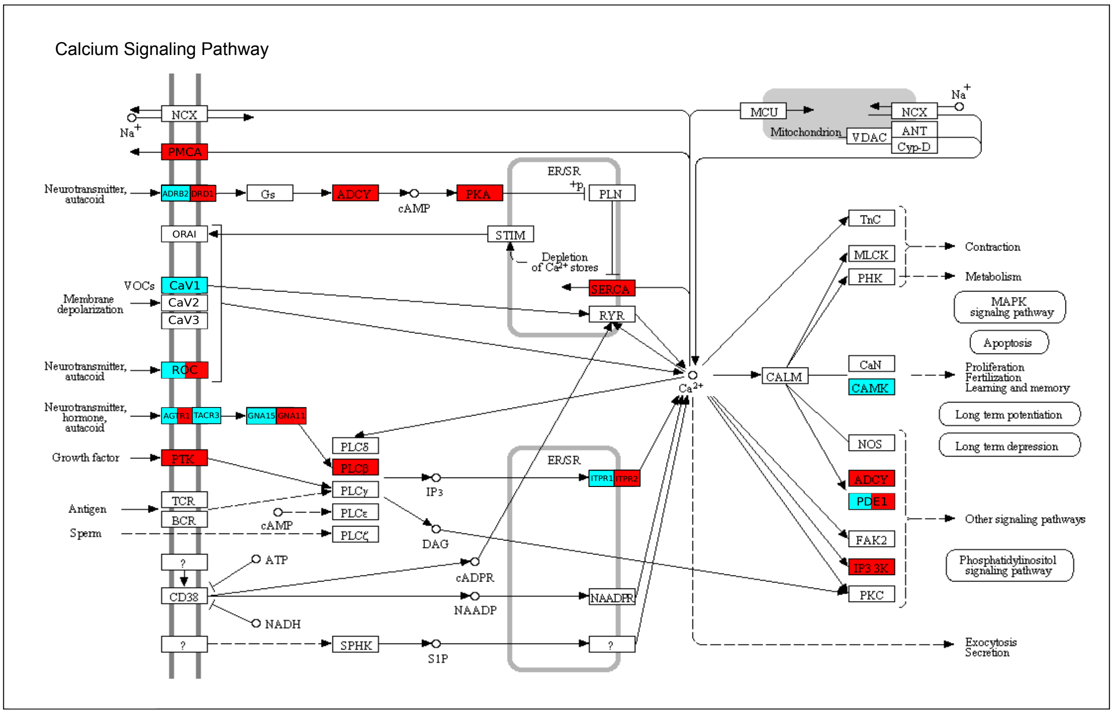

B

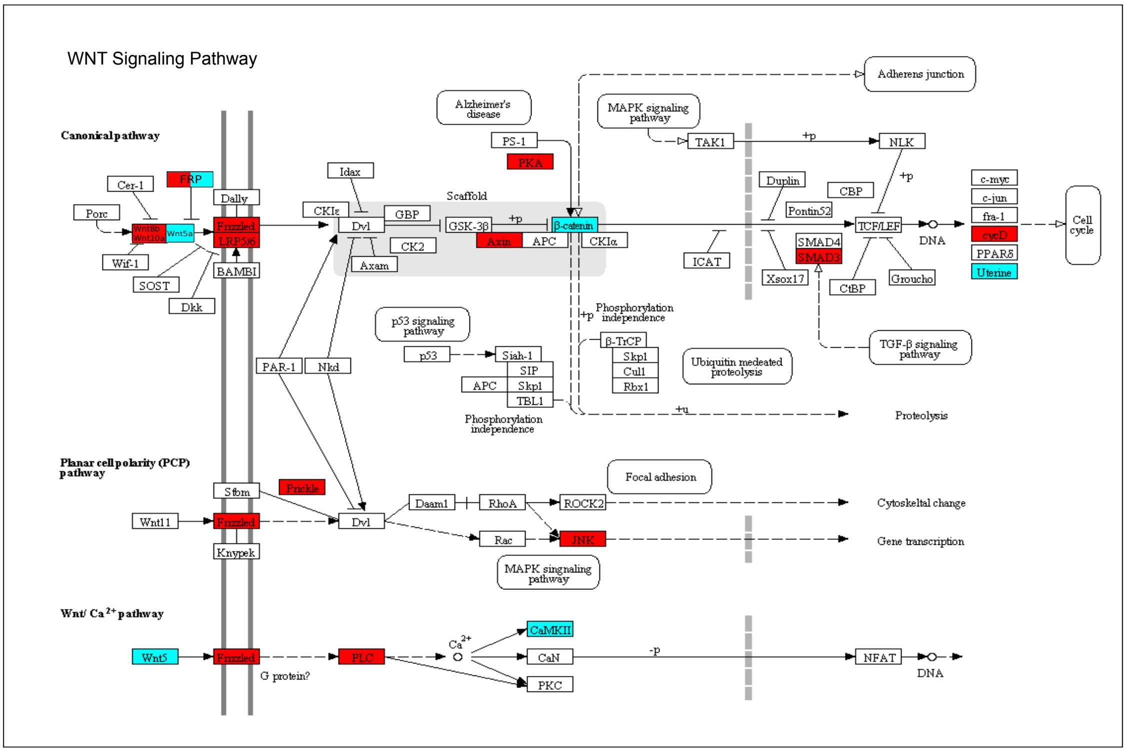

Figure E4. **Assignment of DMRs identified in children of stressed mothers to selected KEGG pathways.** Hypermethylated DMRs are depicted in red, while hypomethylation is shown in blue. Shown are (A) "Calcium signaling pathway", and (B) "Wnt signaling pathway" which we identified as significantly enriched in DMRs.

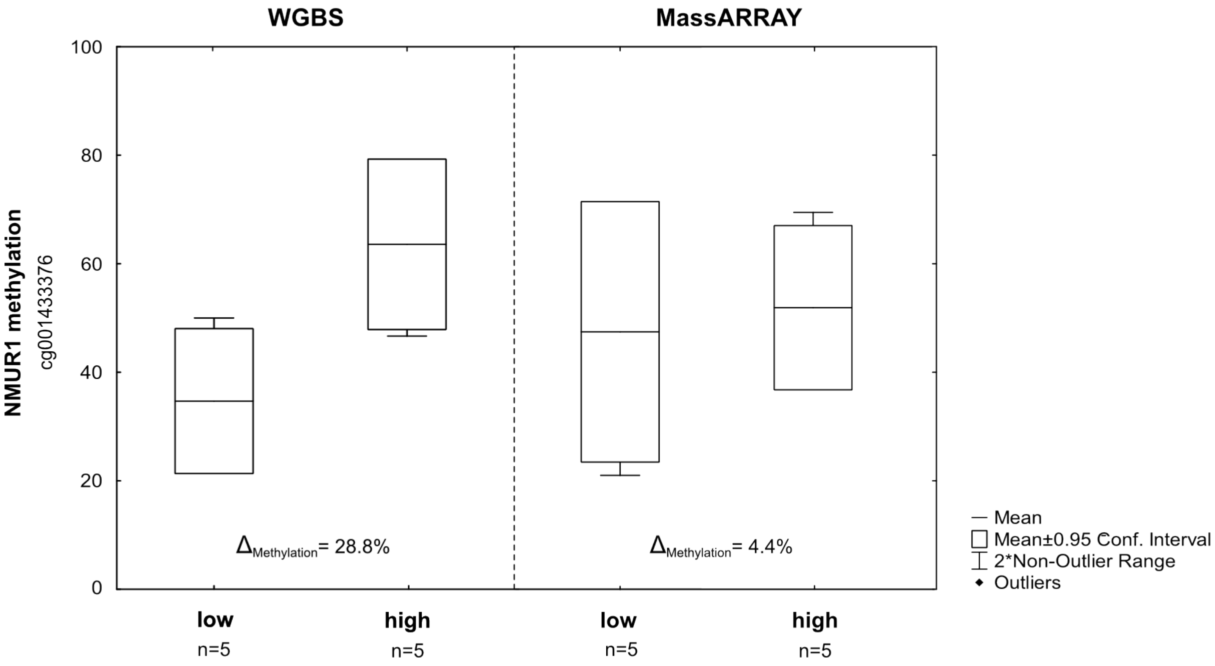

Figure E5. **Comparison of methylation values obtained by WGBS and MassARRAY.** Depicted are methylation values of the first CpG in the detected *NMUR1* DMR determined in the same individuals by WGBS or MassARRAY respectively (only this single position could be considered since the two subsequent CpGs of the DMR yielded a silent peak in the MassARRAY analysis). Given are mean +/- 95% CI, whiskers +/- non-outlier range

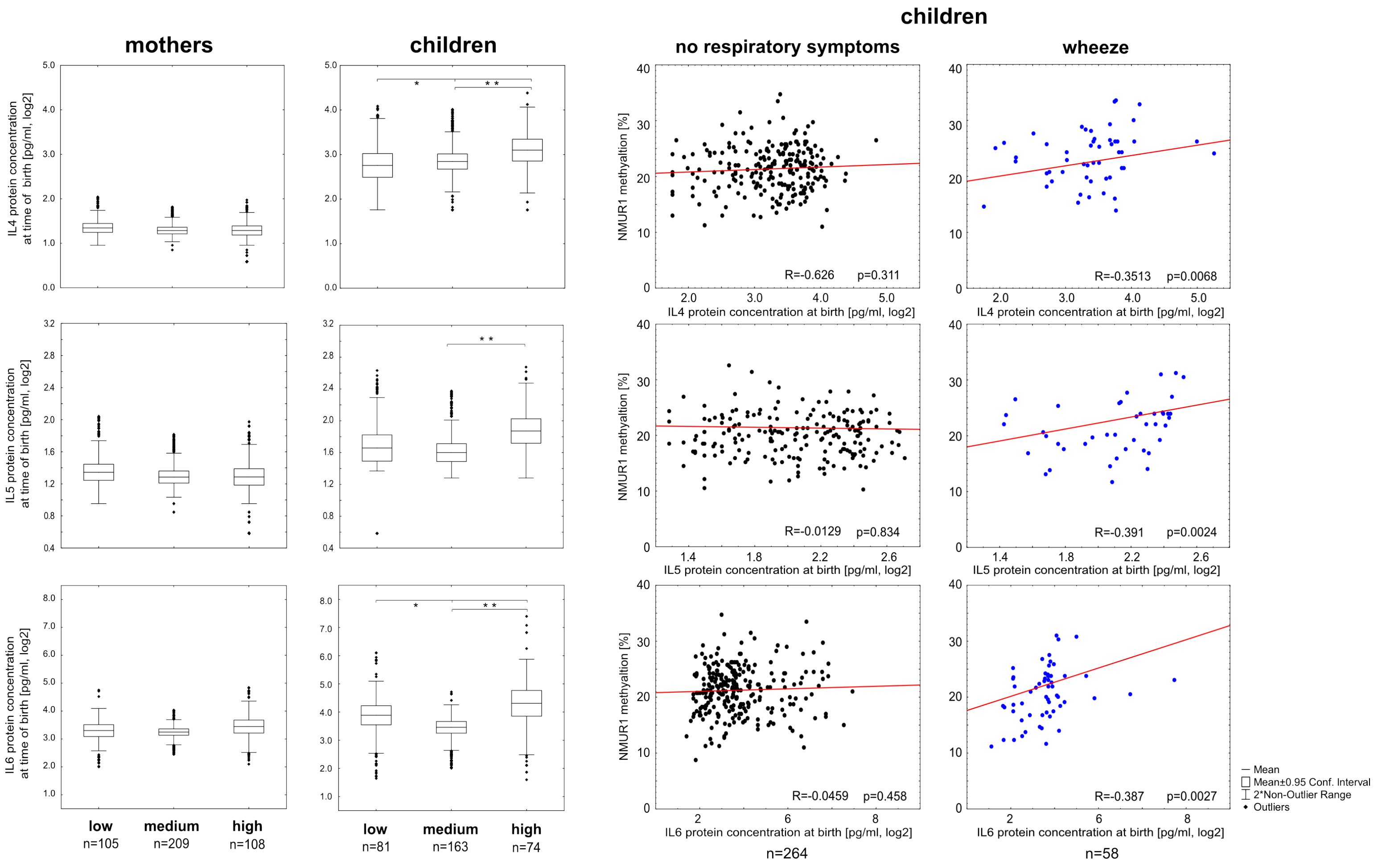

Figure E6. **Elevated Th2 cytokine concentrations in cord blood observed in prenatally stressed children are related to *NMUR1* methylation.** A stress-dependent elevation of IL-4, -5, and -6 concentrations was observed in cord blood while maternal stress did not influence protein concentrations of these cytokines in mothers at time of birth (36th week of gestation). (mean +/- 95% CI, whiskers +/- non-outlier range; p < 0.05, MWU *p<0.05, **p<0.01). A positive correlation between mean methylation in the *NMUR1* enhancer and concentration of all three cytokines was observed in children with late or persistent wheeze, while in non-symptomatic children no such correlation was observed.

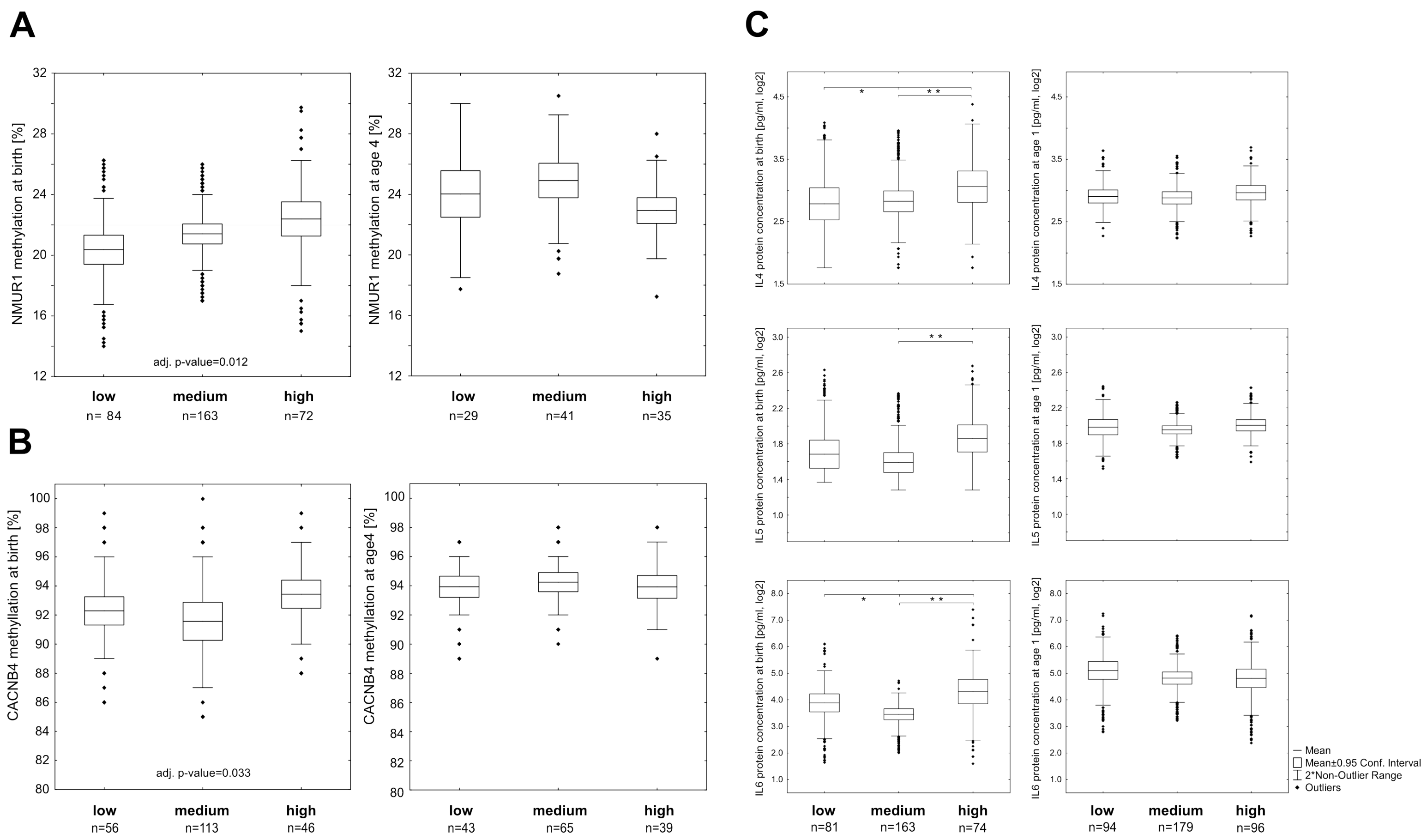

Figure E7. **Prenatal stress induced effects can be transient.** Differential methylation observed at time of birth in the (A) *NMUR1* and (B) *CACNB4* enhancer were no longer observable at age 4. p-values of the multiple regression analysis at time of birth were adjusted for: gender of the child, parental history of atopy, age of the mother, maternal medication and smoking during pregnancy, mode of delivery, birth week and cell composition. (C) Differential cytokine secretion due to prenatal maternal stress observed in children at time of birth had subsided in one-year-old children (mean +/- 95% CI, whiskers +/- non-outlier range; $p < 0.05$, MWU *$p<0.05$, **$p<0.01$).

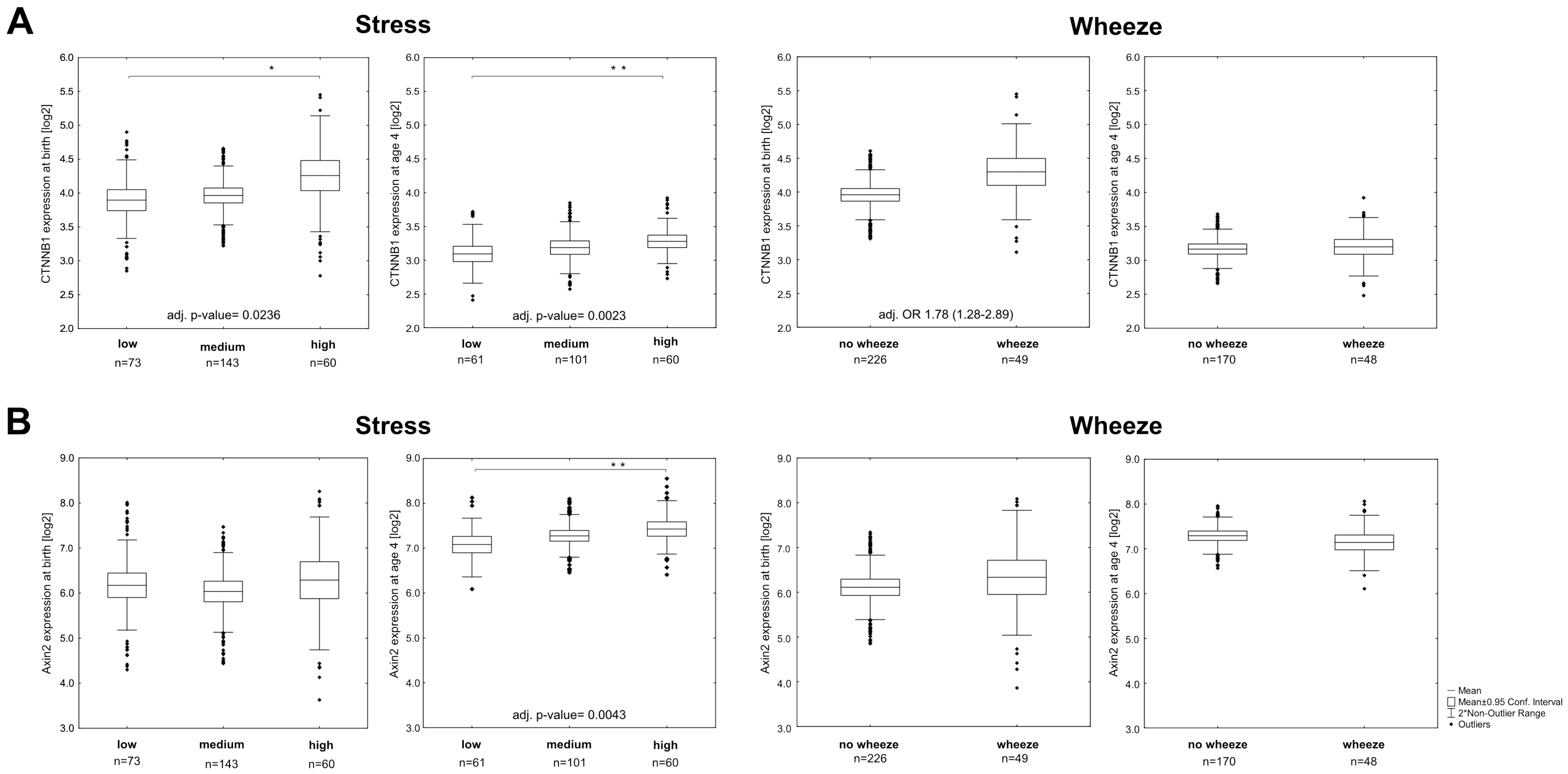

Figure E8. **Canonical Wnt-signaling is perturbed by stress but is not affected in wheezing children at year 4.** Gene expression of (A) β-catenin (*CTNNB1*) and (B) *AXIN2* is significantly higher in four-year-old children exposed to high maternal stress levels during gestation compared to prenatally low stressed children (given are p-values adjusted for: gender of the child, parental atopy, age of the mother, maternal medication and smoking during pregnancy, postnatal ETS exposure, mode of delivery, birth week). Increased expression of *CTNNB1* at time of birth was associated with an increased risk for the child to develop late or persistent wheeze (logistic regression adjusted for. gender of the child, siblings, smoking during pregnancy, ETS exposure after birth, cat keeping, parental history of atopy and parental educational level). No differential transcription was observed in children at year 4 due to the observed phenotype.

A

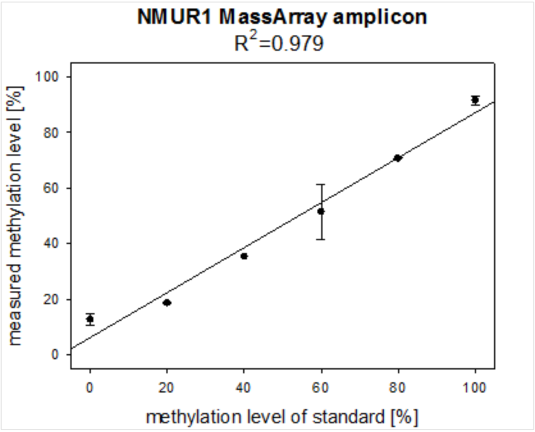

B

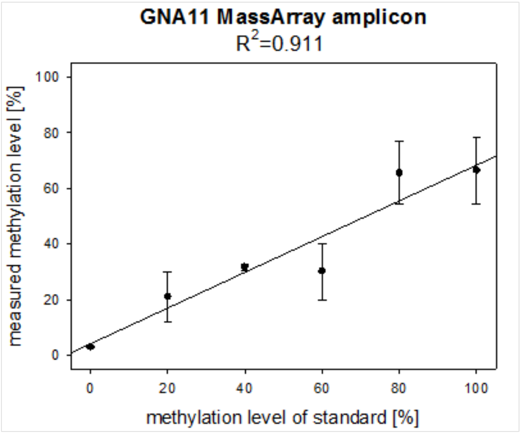

C

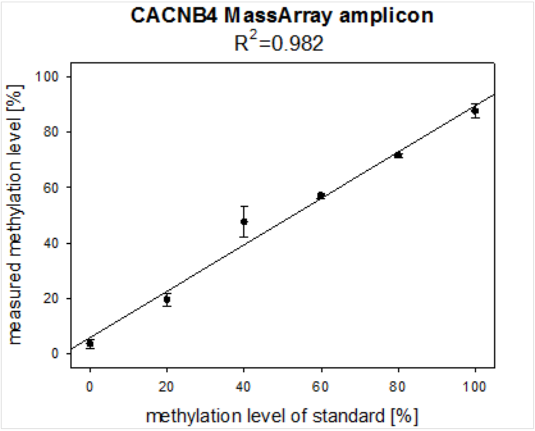

Figure E9. **Methylation values derived for standard samples by MassARRAY.** Depicted are the mean methylation levels over all CpGs within the amplicon-covering regions used for the validation of the (A) NMUR1-, (B) GNA11- and (C) CACNB4-DMRs in the entire cohort (given are the mean methylation values of two independent measurements, bars represent SD).

Table E1

Perceived Stress Questionnaire

	**The following questions contain a series of statements. Please read each statement and select the answer out of the four which states how often the statement is true about you in general.** *(just one cross in each line)*	***hardly ever***	***sometimes***	***often***	***usually***
**1**	You feel rested.	❒	❒	❒	❒
**2**	You have the feeling that too many demands are made of you.	❒	❒	❒	❒
**3**	You have too much to do.	❒	❒	❒	❒
**4**	You feel you are doing things you really like.	❒	❒	❒	❒
**5**	You're worried that you cannot reach your objectives.	❒	❒	❒	❒
**6**	You feel calm.	❒	❒	❒	❒
**7**	You feel frustrated.	❒	❒	❒	❒
**8**	You're full of energy.	❒	❒	❒	❒
**9**	You feel tense.	❒	❒	❒	❒
**10**	Your problems seem to be piling up.	❒	❒	❒	❒
**11**	You feel harassed.	❒	❒	❒	❒
**12**	You feel safe and protected.	❒	❒	❒	❒
**13**	You have lots of worries.	❒	❒	❒	❒
**14**	You're having fun.	❒	❒	❒	❒
**15**	You're afraid for the future.	❒	❒	❒	❒
**16**	You're light-hearted.	❒	❒	❒	❒
**17**	You feel mentally exhausted.	❒	❒	❒	❒
**18**	You have problems relaxing.	❒	❒	❒	❒
**19**	You have enough time for yourself.	❒	❒	❒	❒
**20**	You feel under pressure of time.	❒	❒	❒	❒

Table E2

Summary of discovery cohort and sequencing statistics

Sample ID	Gender	Stress level	Total reads sequenced	Total base pairs sequenced	Average CpG coverage after full processing (chr1-22)
**mothers**					
LMCS00_008m	F	high	1.08E+09	1.09E+11	18.90
LMCS00_020m	F	high	1.17E+09	1.18E+11	20.72
LMCS00_023m	F	high	9.74E+08	9.84E+10	17.01
LMCS00_046m	F	high	1.01E+09	1.02E+11	17.59
LMCS00_047m	F	high	1.15E+09	1.16E+11	21.24
LMCS00_048m	F	low	1.20E+09	1.21E+11	21.87
LMCS00_030m	F	low	1.13E+09	1.14E+11	20.14
LMCS00_019m	F	low	1.09E+09	1.10E+11	18.73
LMCS00_021m	F	low	1.13E+09	1.14E+11	20.29
LMCS00_049m	F	low	1.15E+09	1.16E+11	21.09
**children**					
LMCS00_008c	M	high	1.16E+09	1.17E+11	20.76
LMCS00_020c	M	high	1.10E+09	1.12E+11	20.28
LMCS00_023c	F	high	1.27E+09	1.28E+11	22.47
LMCS00_046c	F	high	9.88E+08	9.98E+10	16.59
LMCS00_047c	F	high	1.15E+09	1.16E+11	21.42
LMCS00_019c	M	low	1.03E+09	1.04E+11	18.46
LMCS00_021c	F	low	1.01E+09	1.02E+11	17.54
LMCS00_030c	F	low	1.14E+09	1.15E+11	20.45
LMCS00_048c	F	low	1.17E+09	1.18E+11	21.89
LMCS00_049c	F	low	1.12E+09	1.14E+11	19.82

Table E3

Comparison of sample characteristics in analysed subcohort versus entire cohort

Parameters	Analysed subcohort				Entire LINA cohort				P value*			
	DNA		RNA		DNA		RNA		DNA		RNA	
	birth n=324	age 4 n=220	birth n=313	age 4 n=227	birth n=469	age 4 n=226	birth n=443	age 4 n=231	birth	age 4	birth	age 4
	%	%	%	%	%	%	%	%	*Chi squared test for crossrelationship sub- vs. entire LINA cohort			
**Gender of the child**												
male	51.5	51.2	50.5	50.7	52.0	50.7	52.4	50.6	0.95	0.99	0.65	0.93
female	48.5	48.8	49.5	49.3	48.0	49.3	47.6	49.4				
**Parental history of atopy**												
negative	32.7	30.9	33.2	32.2	33.5	31.0	34.0	31.9	0.94	0.98	0.87	0.99
single positive	47.5	49.3	49.2	48.5	46.3	48.5	47.3	48.1				
double positive	19.8	19.8	17.6	19.4	20.3	20.5	18.7	20.0				
**Parental education**												
low	1.5	0.5	1.3	0.9	2.8	0.9	2.7	0.9	0.44	0.87	0.39	0.99
intermediate	22.2	25.8	23.0	25.1	23.7	25.8	23.8	24.7				
high	76.2	73.7	75.7	74.0	73.6	73.4	73.6	74.5				
**Pet keeping (cat)**												
no	85.5	84.8	84.7	84.1	85.1	83.0	83.9	83.0	0.96	0.69	0.85	0.83
yes	14.4	15.2	15.3	15.9	14.9	17.0	16.1	17.0				
**Smoking during pregnancy**												
never	87.7	89.4	89.1	89.9	85.3	87.8	86.7	89.4	0.67	0.95	0.63	0.99
occasionally	6.5	5.1	5.4	4.4	6.6	5.7	5.6	4.3				
once a week	0.6	0.5	0.6	0.4	0.6	0.4	0.7	0.4				
daily	5.2	5.1	4.8	5.3	7.5	6.1	7.1	6.0				
**ETS exposure after birth**												
no	94.8	94.0	95.5	94.7	93.3	94.3	94.0	94.4	0.50	0.89	0.46	0.93
yes	5.2	5.5	4.5	5.3	6.7	5.7	6.0	5.6				
**Maternal stress during pregnancy**												
low	25.9	27.6	25.6	26.9	26.0	27.4	26.0	27.3	0.93	1.00	0.87	1.00
medium	51.5	46.1	51.4	45.8	50.4	46.0	49.7	45.5				
high	22.5	26.3	23.0	27.3	23.6	26.5	24.4	27.3				

Table E4

This table is provided as an Excel file, which contains all DMRs (including gDMRs and ngDMRs) identified in children and their mothers and their respective overlap.

Table E5

Enrichment of DMRs in functional regulatory genomic regions as determined by ENCODE

	all DMRs				gDMRs				ngDMRs			
	**Children**		**Mother**		**Children**		**Mothers**		**Children**		**Mothers**	
	number	enrichment	number	enrichment	number	enrichment	number	enrichment	number	enrichment	number	enrichment
**DMRs overlapping with:**												
Promoter*	82	0.91	68	0.99	55	0.1701	40	0.99	27	**3.11E-02**	28	0.06
Enhancer	337	**1.14E-03**	376	**1.98E-05**	250	**5.45E-02**	272	**2.38E-02**	87	**3.97E-05**	104	**2.77E-07**
intergenic	158	**7.55E-04**	180	**3.28E-05**	122	**1.02E-02**	128	**1.18E-02**	36	**1.24E-02**	52	**7.61E-06**
intragenic	179	7.60E-2	197	**3.25E-02**	128	0.53	145	0.24	51	**9.31E-04**	52	**5.78E-03**

* promoter defined as 1000kb down or 2000kb upstream of TSS

Table E6

DMRs associated with genes of relevant KEGG pathways (genes affected by DMRs both in mothers and children in bold)

(A)

	KEGG pathway	Number of DMRs in pathway	p-value	gene symbol								
**Mothers**	**Neuroactive ligand receptor interaction**	34	0.005	ADRA1B<br>CALCR<br>CGA<br>CHRM5	**CNR1**<br>F2RL2<br>FPR1<br>**FSHR**	GABRP<br>**GALR1**<br>**GLRA1**<br>GRIA1	GRIN2A<br>GRIN2B<br>**GRM7**<br>HTR1A	HTR1B<br>HTR1F<br>HTR2A<br>**LEPR**	LPAR1<br>**MC2R**<br>**MC4R**<br>NMBR	NPBWR2<br>**P2RX2**<br>P2RY1<br>PARD3	PTGIR<br>RXFP1<br>S1PR4<br>**TACR3**	TRHR<br>**VIPR2**
**Children**	**Neuroactive ligand receptor interaction**	35	0.005	ADRB2<br>AGTR1<br>CHRNA5<br>**CNR1**	DRD1<br>DRD3<br>F2RL3<br>**FSHR**	GABRB1<br>GABRB3<br>**GALR1**<br>GHSR	**GLRA1**<br>GRIA3<br>GRIA4<br>GRIK2	GRIK4<br>**GRM7**<br>**LEPR**<br>**MC2R**	**MC4R**<br>MC5R<br>MCHR1<br>MCHR2	NMUR1<br>NMUR2<br>**P2RX2**<br>P2RY8	PRSS3<br>PTH2R<br>RXFP1<br>RXFP2	**TACR3**<br>TSPO<br>**VIPR2**
	**Calcium signaling pathway**	19	0.039	ADCY1<br>ITPKB<br>GNA15	ADRB2<br>ITPR1	AGTR1<br>ITPR2	ATP2A3<br>P2RX2	CACNA1S<br>PDE1C	CAMK2B<br>PLCB4	DRD1<br>PRKX	ERBB2<br>TACR3	GNA11<br>ATP2B2
	**Wnt signaling pathway**	20	0.004	AXIN1<br>PLCB4<br>MMP7	AXIN2<br>PPP2R5A<br>LRP6	CAMK2B<br>PRICKLE1	CCND2<br>PRKX	CTNNB1<br>SFRP1	FZD5<br>SMAD2	FZD8<br>SMAD3	WNT10A<br>WNT8B	MAPK9<br>WNT5A

(B) Overlapping DMRs in mothers and children.

		chr	start	end	CpGs in DMR	Δ mean stress score 2 vs. 0	p-value	annotation	nearest promoter ID	gene name
**Neuroactive ligand receptor interaction**	children	7	158882705	158882828	5	-0.229	0.007	intron	NM_003382	VIPR2
	mothers	7	158882741	158882828	4	-0.199	0.012	intron	NM_003382	VIPR2
	children	4	104749071	104749203	3	-0.376	0.005	intergenic	NM_001059	TACR3
	mothers	4	104749071	104749203	3	-0.404	0.002	intergenic	NM_001059	TACR3
	children	12	133201860	133202133	10	-0.210	0.025	intron	NM_012226	P2RX2
	mothers	12	133201860	133202011	9	-0.218	0.030	intron	NM_012226	P2RX2
	children	3	6683538	6683638	4	-0.220	0.037	intergenic	NM_181874	GRM7
	mothers	3	6683538	6683638	4	-0.212	0.026	intergenic	NM_181874	GRM7
	children	6	88817586	88817933	3	0.330	0.005	intergenic	NM_033181	CNR1
	mothers	6	88817586	88817933	3	0.322	0.032	intergenic	NM_033181	CNR1

Table E7

qPCR primer pairs

	gene name	forward primer	reverse primer
**Stress axis**	*NMUR1*	cggagacaagtgaccaagatg	cacgacgctccacatgac
	NPY	gctgcgacactacatcaacc	gctttctctcatcaagaggtctg
	NGF	tccggacccaataacagttt	catggacattacgctatgcac
	VIPR2	gatttcgtcgatgcctgtg	ccttcaccagaatataaaacgtga
	NTRK1	ctgcctgagcaggacaagat	actctcggacgcctcctt
**Ca^{2+}signaling**	*CACNB4*	cctcctcctacgccaagaa	gctgccatcggatcttttc
	GNA11	gcatccaggaatgctacgac	ggtcaacgtcggtcaggtag
	PLCB4	acgtcagtccaatgacatgc	ggaattttaccatttgtgttggt
	CACNB2	gcagctgataaactggctca	tcaagctggttctcatccaa
**Wnt signaling**	*AXIN2*	gctgacggatgattccatgt	actgcccacacgataaggag
	CTNNB1	tgttaaattcttggctattacgaca	ccaccactagccagtatgatga
	FZD5	tggagctgcgctaatcct	ttggtgtgtgatccatgagg
	PPP2R5A	tggtgttgctgaacttcttga	gcagtatgcataggaataagaacct
	WNT10A	atccacgcgagaatgagg	ccgcatgttctccatcact
**Downstream targets**	*NFATC3*	ccagcaggaagatatctgagga	ccagggactcaggactcg
	PIK3R1	aatgaacgacagcctgcac	ccgttgttggctacagtagtagg
	PPP3R1	ttgtagacaaaaccataataaatgcag	ggatatctaggccacctacaaca
	PPP3CA	gatgcgccagtcactgttt	ccctaagaagaggtagcgagtg
	CAMK2D	gtcaccaacagtacccatcaac	ttcatgcactcagaaacatgc
**Reference genes**	*GUSB*	cgccctgcctatctgtattc	tccccacagggagtgtgtag
	GAPD	gctctctgctcctcctgttc	acgaccaaatccgttgactc
	PGK1	tgcaaaggccttggagag	tggatcttgtctgcaactttagc
	PPIA	catggtggctcactgtctgt	ggctgatcttgactcctaccc

References

1 Martinez, F. D. *et al.* Asthma and wheezing in the first six years of life. The Group Health Medical Associates. *N Engl J Med* **332**, 133-138, doi:10.1056/nejm199501193320301 (1995).

2 Johnson, M. D., Mueller, M., Game, L. & Aitman, T. J. Single nucleotide analysis of cytosine methylation by whole-genome shotgun bisulfite sequencing. *Current protocols in molecular biology / edited by Frederick M. Ausubel ... [et al.]* **Chapter 21**, Unit21.23, doi:10.1002/0471142727.mb2123s99 (2012).

3 Li, H. & Durbin, R. Fast and accurate short read alignment with Burrows-Wheeler transform. *Bioinformatics (Oxford, England)* **25**, 1754-1760, doi:10.1093/bioinformatics/btp324 (2009).

4 Cirulli, F. & Alleva, E. The NGF saga: from animal models of psychosocial stress to stress-related psychopathology. *Frontiers in neuroendocrinology* **30**, 379-395, doi:10.1016/j.yfrne.2009.05.002 (2009).

5 Hansen, K. D., Langmead, B. & Irizarry, R. A. BSmooth: from whole genome bisulfite sequencing reads to differentially methylated regions. *Genome Biol* **13**, R83, doi:10.1186/gb-2012-13-10-r83 (2012).

6 Tusher, V. G., Tibshirani, R. & Chu, G. Significance analysis of microarrays applied to the ionizing radiation response. *Proc Natl Acad Sci U S A* **98**, 5116-5121, doi:10.1073/pnas.091062498 (2001).

7 Schwender, H. siggenes: Multiple testing using SAM and Efron's empirical Bayes approaches, R package version 1.36.0. (2012).

8 Quinlan, A. R. & Hall, I. M. BEDTools: a flexible suite of utilities for comparing genomic features. *Bioinformatics (Oxford, England)* **26**, 841-842, doi:10.1093/bioinformatics/btq033 (2010).

9 *Database of Single Nucleotide Polymorphisms (dbSNP). Bethesda (MD): National Center for Biotechnology Information, National Library of Medicine. (dbSNP Build ID: {141})*, <http://www.ncbi.nlm.nih.gov/SNP/> (

10 Liu, Y., Siegmund, K. D., Laird, P. W. & Berman, B. P. Bis-SNP: combined DNA methylation and SNP calling for Bisulfite-seq data. *Genome Biol* **13**, R61, doi:10.1186/gb-2012-13-7-r61 (2012).

11 Consortium, E. P. An integrated encyclopedia of DNA elements in the human genome. *Nature* **489**, 57-74, doi:10.1038/nature11247 (2012).

12 Rosenbloom, K. R. *et al.* ENCODE data in the UCSC Genome Browser: year 5 update. *Nucleic acids research* **41**, D56-63, doi:10.1093/nar/gks1172 (2013).

13 Roadmap Epigenomics, C. *et al.* Integrative analysis of 111 reference human epigenomes. *Nature* **518**, 317-330, doi:10.1038/nature14248 (2015).

14 Ehrich, M. *et al.* Cytosine methylation profiling of cancer cell lines. *Proceedings of the National Academy of Sciences of the United States of America* **105**, 4844-4849, doi:10.1073/pnas.0712251105 (2008).

15 Reinius, L. E. *et al.* Differential DNA methylation in purified human blood cells: implications for cell lineage and studies on disease susceptibility. *PloS one* **7**, e41361, doi:10.1371/journal.pone.0041361 (2012).

16 Jaffe, A. E. & Irizarry, R. A. Accounting for cellular heterogeneity is critical in epigenome-wide association studies. *Genome biology* **15**, R31, doi:10.1186/gb-2014-15-2-r31 (2014).

17 Pascual, M. *et al.* Epigenetic changes in B lymphocytes associated with house dust mite allergic asthma. *Epigenetics : official journal of the DNA Methylation Society* **6**, 1131-1137, doi:10.4161/epi.6.9.16061 (2011).

18 Pei, L. *et al.* Genome-wide DNA methylation analysis reveals novel epigenetic changes in chronic lymphocytic leukemia. *Epigenetics : official journal of the DNA Methylation Society* **7**, 567-578, doi:10.4161/epi.20237 (2012).

19 Livak, K. J. & Schmittgen, T. D. Analysis of relative gene expression data using real-time quantitative PCR and the 2(-Delta Delta C(T)) Method. *Methods* **25**, 402-408, doi:10.1006/meth.2001.1262 (2001).
20 Herberth, G. *et al.* Association of neuropeptides with Th1/Th2 balance and allergic sensitization in children. *Clin Exp Allergy* **36**, 1408-1416, doi:10.1111/j.1365-2222.2006.02576.x (2006).
